# Quantitative Proteomics Unveils the Synergistic Effects of Combination Drugs on Cytoskeleton Composition and Autophagy-Mediated Cell Death in Neuroblastoma

**DOI:** 10.1021/acs.jproteome.5c00191

**Published:** 2025-06-17

**Authors:** Pei-Chen Yu, Yi-Chun Kao, Hsin-Yi Chang, Chen-Hao Huang, Wen-Ming Hsu, Hsuan-Cheng Huang, Hsueh-Fen Juan

**Affiliations:** † Institute of Molecular and Cellular Biology, 33561 National Taiwan University, Taipei 106, Taiwan; ‡ Department of Life Science, National Taiwan University, Taipei 106, Taiwan; § Graduate Institute of Medical Sciences, 71548 National Defense Medical Center, Taipei 114, Taiwan; ∥ Graduate Institute of Biomedical Electronics and Bioinformatics, National Taiwan University, Taipei 106, Taiwan; ⊥ Department of Surgery, 38006 National Taiwan University Hospital and National Taiwan University College of Medicine, Taipei 100, Taiwan; # Institute of Biomedical Informatics, National Yang Ming Chiao Tung University, Taipei 112, Taiwan; ¶ Center for Computational and Systems Biology, National Taiwan University, Taipei 106, Taiwan

**Keywords:** neuroblastoma, pyrvinium pamoate, sirolimus, tandem mass tag, combination therapy

## Abstract

Neuroblastoma, a prevalent and aggressive childhood cancer, lacks effective treatments. Recent research highlights the repurposing of existing drugs as a strategy for breakthroughs in combating this disease. We systematically analyzed small-molecule perturbation gene expression data from the Library of Integrated Network-Based Cellular Signatures (LINCS), identifying pyrvinium pamoate and sirolimus, two FDA-approved drugs, as potential candidates for neuroblastoma combination therapy. Colony formation assays and organoid culture confirmed that the therapeutic effect of combining these two drugs exceeded that of either drug alone. The mRNA expression levels of several genes predicted by LINCS also decreased. To comprehensively understand the mechanism behind superior efficacy of the combination therapy compared to monotherapy, we performed quantitative proteomics with tandem mass tag labeling and identified 3416 proteins from 20,623 peptides. Gene set enrichment analysis and Database for Annotation, Visualization, and Integrated Discovery revealed that combination therapy significantly decreased cytoskeleton formation compared with monotherapy, reflecting dramatic reduction in cell migration. Additionally, the research indicated that cell cycle arrest occurred under combination therapy. Furthermore, we confirmed that the extent of autophagy significantly increased after the combination treatment. In summary, this study elucidates the mechanisms and therapeutic potential of combining sirolimus and pyrvinium pamoate for treating neuroblastoma, offering new advancements for this challenging disease.

## Graphical abstract

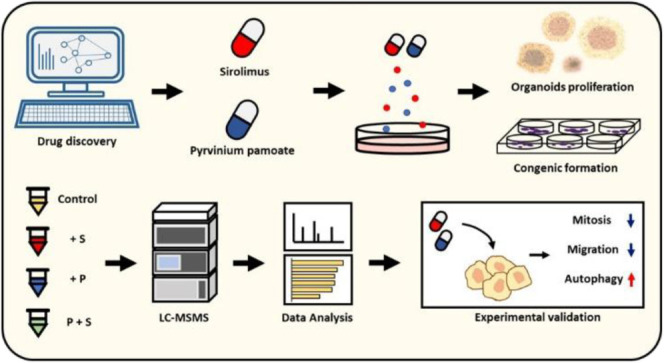

## Introduction

Neuroblastoma is the most common solid tumor in children and ranks as the third most prevalent pediatric cancer overall, following leukemia and brain cancer.^1^ This malignant tumor originates from the sympathetic nervous system, typically starting in the adrenal gland.^2^ It can also metastasize to other areas, such as the chest, abdomen, bones, liver, and skin. Symptoms vary depending on the tumor’s location and extent of metastasis and may include fatigue, fever, weight loss, loss of appetite, diarrhea, irritability, and memory loss, with additional signs like skin discoloration and bone pain.^3^ The cause of neuroblastoma is largely unknown, and while most cases are nonhereditary, a few are hereditary. Current treatment options include surgical resection, chemotherapy, radiation therapy, and immunotherapy. Children under 18 months with low-risk disease may experience spontaneous regression without therapeutic intervention.^4^ In contrast, high-risk neuroblastoma typically occurs in children over 18 months and often involves *MYCN* amplification;^5^ approximately half of these patients experience relapse. Despite combination therapies, the 5 year survival rate for high-risk patients remains at 40% to 50%.^6^ Therefore, developing novel and more effective treatments is a critical priority.

Combination therapy, which has gained prominence in recent years, can potentially reduce dosage and side effects while minimizing the development of drug resistance.^7^ This approach is increasingly seen as a promising therapeutic strategy. Historically, cancer research has focused on investigating physiological pathways and clinical treatments. However, the advent of high-throughput technologies has led to a surge in cancer-related data.^8^ Major initiatives like The Cancer Genome Atlas in the United States and the International Cancer Genome Consortium exemplify this trend.^9^ With the rise of bioinformatics, this field has become essential for integrating these data and applying various statistical modeling techniques to explore the relationships between cancer and genes. These insights are then utilized in new drug development, drug repurposing, and other therapeutic applications. For instance, Crizotinib was developed through a structure-based drug design approach, and medicinal chemistry leads optimization as part of Pfizer’s extensive SAR program.^10^ Our study employed a drug screening method^11^ to identify potential candidates for combination therapy in neuroblastoma and to further investigate their efficacy.

The drug we have chosen, sirolimus, targets the mammalian target of rapamycin (mTOR), a serine/threonine protein kinase that regulates cell growth, proliferation, movement, metabolism, and survival in mammals.^12,13^ In mammals, mTOR forms two protein complexes, known as mTOR Complex 1 (mTORC1) and mTOR Complex 2 (mTORC2).^14^ mTORC1 is hyperactivation in several human cancers, including hepatocellular carcinoma,^15^ esophageal squamous cell carcinoma,^16^ and neuroblastoma.^17^ The overexpression of mTOR1 downstream effectors such as 4E-BP1, S6K, and eIF4E is highly correlated with poor prognosis in these cancers.^18–20^ mTOR2 regulates tumor cell initiation and development,^21^ and RAS mutations enhance mTORC2 kinase activity, triggering downstream cell cycle and transcriptional program.^22^ Additionally, mTOR is associated with drug resistance in various cancers including gastric cancer, nonsmall cell lung cancer, and melanoma.^23,24^

The other drug that we selected is pyrvinium pamoate, which targets the WNT signaling pathway. The pathway is crucial in numerous biological processes such as embryonic development, cell proliferation, self-renewal, and differentiation.^25^ Activation of the canonical WNT signaling pathway leads toβ-catenin-mediated transcriptional changes.^26^ Components of the canonical WNT signaling pathway are integral to the pathology of various cancers^27^ including colorectal cancer,^28^ brain tumors,^29^ melanoma,^30^ breast cancer,^31^ liver,^32^ and pancreatic cancer,^33^ with WNT pathway proteins localized to the cell membrane, cytoplasm, and nucleus. Therefore, the mTOR and WNT signaling pathway may offer novel targets for neuroblastoma treatment.^34^

To delve deeper into the potential impact of combination treatments, we utilized proteomics to examine the differential protein expression between treatments. Proteomics is a burgeoning field in cancer research and has garnered significant attention recently. The most widely used technique for protein identification and quantification is liquid chromatography coupled with tandem mass spectrometry (LC–MS/MS).^35^ In this method, proteins from biological samples are separated and enzymatically digested into peptides, which are then analyzed by mass spectrometry. The acquired MS1 and MS2 spectra were matched to the theoretical ones to identify the peptide sequences.^36^ Currently, one of the most commonly used sample labeling methods is tandem mass tag (TMT), an isobaric tagging technique capable of labeling up to 18 samples or more simultaneously. TMT labeling facilitates multiplexing, allowing for the simultaneous analysis of multiple samples, thereby reducing experimental variability inherent in sample processing.^37^ Our study employs the TMT labeling method to investigate the differential protein expression between various treatments.

The findings of our study illuminate the promising potential of repurposing existing drugs for innovative therapeutic combinations. Combining sirolimus and pyrvinium pamoate has not only demonstrated significant inhibitory effects on neuroblastoma growth but also unveiled a novel path for targeted therapy against this challenging cancer. This research demonstrates the practical application of bioinformatics and proteomics in identifying and validating a new drug combination of sirolimus and pyrvinium pamoate, offering a novel therapeutic approach for treating neuroblastoma.

## Materials and Methods

### Cell Lines

The neuroblastoma cell lines SK-N-BE-(2)­C and SK-N-DZ were purchased from the American Type Culture Collection. Meanwhile, the SK-N-AS and SK-N-SH cell lines were obtained from Yung-Feng Liao at the Institute of Cellular and Organismic Biology, Academia Sinica. The cells were cultured in Dulbecco’s modified Eagle’s medium (DMEM; Gibco Laboratories) supplemented with 10% fetal bovine serum (FBS; Gibco Laboratories) under conditions of 5% CO_2_ and 37 °C.

### Cell Viability Assay

Cells were seeded in a 96-well plate at a density of 5000 cells per well for 24 h and treated with different concentrations of drugs: 0, 10, 20, and 40 μM for sirolimus (MedChemExpress) or 0.3125, 0.625, 1.25, 2.5, 5, and 10 μM for pyrvinium pamoate (MedChemExpress). Cell viability was assessed using the CellTiter-Glo Luminescent Cell Viability Assay kit (Promega Corporation) for sirolimus and the MTS assay (Promega Corporation) for pyrvinium pamoate. For the CellTiter-Glo assay, the medium was replaced with fresh medium, cells were lysed with reagent, and after shaking for 10 min in the dark, 180 μL of the solution was transferred to a new 96-well plate for luminescence detection. For the MTS assay, 20 μL of MTS reagent was added to each well, and the plate was incubated for 2 h at 37 °C with 5% CO_2_. Absorbance was measured at 490 nm by using a microplate reader (Molecular Devices). The values were background-subtracted and normalized to the control (DMSO) group. The IC_50_ was calculated for each treatment.

### Colony Forming Assay

Cells were seeded onto 6-well plates at a density of 1 × 10^3^ cells per well and allowed to adhere for 2 days. Cells were treated with sirolimus and pyrvinium pamoate at the indicated doses. The medium containing the drugs was replaced every 3 days, and the cells were cultured for a total of 14 days to allow colony formation. The colonies were washed twice with PBS, fixed with methanol at room temperature overnight, and then stained with 1% crystal violet for 16 h.

### Drug Response Quantification and Synergy Analysis

Cell viability (%) for each drug combination was determined using colony formation assays. The inhibition rate was calculated using the following formula:

Inhibition = 100 – viability.

The resulting data were structured into a matrix using R (version 4.3.1) with rows and columns corresponding to the concentrations of Drug S and P, respectively.

To evaluate the potential drug synergy, we applied the Zero Interaction Potency model,^38^ which assumes that the drugs do not interact and serve as independent agents. The expected inhibition for a given drug combination was calculated as

ZIPij=Ii0×I0j/100

where *Ii*0 and *I*0*j* represent the inhibition rates of Drug S and Drug P alone at concentrations *i* and *j*, respectively. *Iij* denotes the observed inhibition rate when both drugs are used in combination at the same concentrations. The delta synergy score was then calculated as the difference between the observed inhibition and the expected inhibition values

Δij=Iij−ZIPij

A positive delta score (Δ*ij* >0) indicates synergy, a score near zero indicates additivity, and a negative value suggests antagonism. All analyses and heatmap visualizations were performed using the ggplot2 (version 3.4.2) and reshape2 (version 1.4.4) packages. Delta scores were visualized in a heatmap, with color gradients representing interaction types, blue for antagonism and orange for synergy, and each cell annotated with its corresponding delta value.

### Patient Material Processing

The patient specimens are washed twice with PBS solution (containing Primocin 1 μg/mL, ant-pm-1, InvivoGen). The specimens are then placed in an organoid culture medium (200 mL of Neurobasal Medium, Thermo Fisher Scientific; 250 mL of serum-free DMEM/F-12 GlutaMAX supplement, Thermo Fisher Scientific; 2.5 mL of N-2 Supplement (100X), Thermo Fisher Scientific; 5 mL of B-27 Supplement (50×), Thermo Fisher Scientific; and 25 mL of FBS, Gibco Laboratories), with the addition of 500 μg/mL Collagenase I (Worthington) and 500 μg/mL Collagenase IV (Worthington). The specimens are then cut into 1–2 mm^3^ pieces using sterile scissors. The enzymatic digestion is carried out at 37 °C for 60 min. If undigested fragments remain after 60 min, digestion is continued for an additional 30 min until all fragments are digested. The resulting cell suspension is filtered through a 70 μm filter. The filtrate is then centrifuged at 1200 rpm for 5 min at 4 °C, and the supernatant is discarded. The pellet is resuspended in the organoid culture medium (containing 20 ng/mL Human FGF-basic (FGF-2/bFGF) Recombinant Protein, 13256-029, Thermo Fisher Scientific; 20 ng/mL Human EGF Recombinant Protein, PHG0311, Thermo Fisher Scientific) and cultured at 37 °C with 5% CO_2_. This sample was approved by the Ethics Review Committee of the College of Medicine, National Taiwan University (202311011RIND), and all research participants provided written informed consent.

### Isolation of Specimen Cells

The cell suspension obtained from the digestion of the patient specimens is centrifuged at 1200 rpm, 4 °C for 5 min. After centrifugation, the supernatant is removed, and the pellet is washed once with PBS. The suspension is then centrifuged again at 1200 rpm and 4 °C for 5 min, and the PBS is discarded. The cells are then resuspended at a concentration of 1 × 10^7^ cells per mL in FC buffer (0.5% BSA, 1 μL/mL Human BD Fc Block, 564219, BD Pharmingen, prepared in wash buffer; the wash buffer contents are 8 g of NaCl, 0.2 g of KCl, 1.44 g of Na_2_HPO_4_, 0.24 g of KH_2_PO_4_, and 0.9 g of sodium azide in 1L of ddH_2_O) and incubated at room temperature for 10 min. For each sample tube of cell suspension, 1 × 10^6^ cells in a final volume of 100 μL are stained (50 μL of cells at a concentration of 100 × 10^6^ cells/mL, with 50 μL of antibody mixture). We use CD45 (PE Anti-CD45 antibody, ab134202, Abcam) and CD56 (APC CD-NCAM antibody (monoclonal), ab28335, Abcam) for cell sorting and use Live/Dead cell stain (LIVE/DEAD Fixable Blue Dead Cell Stain, L34962, Thermo Fisher Scientific) to identify live cells. The antibody mixture conjugated with fluorescent dyes is incubated with the cells on ice, protected from light, for 30 min. After staining, the cells are centrifuged at 300 g, 4 °C for 3 min, and the supernatant is removed. The pellet is resuspended in wash buffer to a final volume of 200 μL. Finally, the cells are sorted using a BD Influx cell sorter, and an index sorting mode is employed to record the fluorescence parameters associated with each sorted cell.

### 3D Organoid Formation and Maintenance

We used the ClinoStar 3D Culture System (CelVivo) for 3D organoid formation. ClinoReactors (CelVivo) for spheroid propagation were humidified with sterile water at room temperature for 4 h before use. Then, 2 mL of organoid medium was added to the culture tank of ClinoReactors and rotated and equilibrated at 15 rpm, 5% CO_2_, and 37 °C for 2 h. After equilibration, the culture medium was removed from the culture tank, cells selected by flow cytometry were transferred to the ClinoReactors at 1 × 10^5^ cells using a syringe, the culture tank was filled with organoid medium 10 mL and cultured in ClinoStar at 15 rpm, 5% CO_2_, and 37 °C for 7 days, and organoids were generated. 90% of the volume of the medium was renewed twice a week until the organoids grew to a diameter of 100 μm. The organoids were then transferred from the culture tank to 10 cm (uncoated) culture dishes and cultured in an environment of 5% CO_2_ and 37 °C. 90% of the culture medium volume was renewed twice a week. When the organoids grow to a diameter of 600–650 μm, proceed to the next experiment.

### Effects of Drug Treatment on Survival of 3D Organoids

Matrigel (Corning) was coated on a 12-well culture plate at 400 μl per well, and 1 mL of organoid medium was placed in each well. Then, 3 organoids with a diameter of 600–650 μm were inoculated into the coated 12-well plate, cultured for 24 h, and treated with different concentrations of drugs: 20 μM sirolimus (MedChemExpress) or 2 μM pyrvinium pamoate (MedChemExpress), and then morphological observations after drug treatment 0, 24, 48, and 72 h were performed with three biological replicates.

### Cell Synchronization

SK-N-AS, SK-N-BE-(2)­C, SK-N-DZ, and SK-N-SH cells were seeded in 12-well plates at a density of 1 × 10^5^ cells per well and incubated for 24 h. After this period, thymidine (Sigma-Aldrich) was added to the medium to achieve a final concentration of 2 mM, and the cells were cultured at 37 °C for 18 h. Subsequently, thymidine was removed by washing the cells with PBS, and fresh medium was added. The cells were then incubated for an additional 9 h at 37 °C. A second round of thymidine was added to a final concentration of 2 mM, and the cells were cultured for another 18 h at 37 °C. This process synchronized the cells at the G1/S boundary. Throughout the synchronization process, the cells were grown in DMEM supplemented with 10% FBS under a 5% CO_2_ and 37 °C.

### Immunofluorescence

For staining to observe mitotic cells, the synchronized cells were treated with 10 μM sirolimus, 5 μM pyrvinium pamoate, or DMSO for 8 h. The cells were fixed in 3.7% paraformaldehyde (Sigma-Aldrich) for 15 min and then sealed in PBS with 5% bovine serum albumin (BSA; BioShop) and 0.2% Triton X-100 (Sigma-Aldrich) for 1 h. The cells were then incubated with the primary antibody: α-tubulin (GTX628802, 1:500, GeneTex), overnight at 4 °C in 5% BSA. Subsequently, FITC or rhodamine-coupled secondary antibodies (A-10680, 1:1000, Thermo Fisher Scientific) were added. The slides were counterstained with DAPI (P36931, Thermo Fisher Scientific) and examined by using an Olympus microscope (Watford). For each treatment group, two large fields were randomly selected for observation (each large field contains nine smaller fields), and three biological replicates were performed.

For cytoskeleton staining, the synchronized SK-N-DZ cells were treated with 16 μM sirolimus, 1 μM pyrvinium pamoate, or DMSO for 24 h. The cells were fixed in 3.7% paraformaldehyde (Sigma-Aldrich) for 15 min and then sealed them in PBS with 5% bovine serum albumin (BSA; BioShop) and 0.2% Triton X-100 (Sigma-Aldrich) for 1 h. The cells were then incubated with the primary antibody ACTB (GTX629630, 1:500, GeneTex) or TUBA1A (GTX112141, 1:500, GeneTex) overnight at 4 °C in 5% BSA. Subsequently, FITC or rhodamine-coupled secondary antibodies (A-10680 and A-21428, 1:1000, Thermo Fisher Scientific) were added. The slides were counterstained with DAPI (P36931, Thermo Fisher Scientific) and examined using an Olympus microscope (Watford). For each treatment group, three fields were randomly selected for observation, and three biological replicates were performed.

For autophagosome staining, the synchronized SK-N-DZ cells were treated with 16 μM sirolimus, 1 μM pyrvinium pamoate, or DMSO in the presence of 30 μM chloroquine diphosphate (L10382, Thermo Fisher Scientific) for 24 h. The cells were fixed in 3.7% paraformaldehyde (Sigma-Aldrich) for 15 min and then sealed in PBS with 5% BSA (BioShop) and 0.2% Triton X-100 (Sigma-Aldrich) for 1 h before incubating them with the primary antibody: LC3B (L10382, 1:2000, Thermo Fisher Scientific) overnight at 4 °C in 5% BSA. FITC secondary antibodies (A-11008, 1:1000, Thermo Scientific) were added and washed three times with PBST. Slides were then counterstained with DAPI (P36931, Thermo Fisher Scientific) and examined using an Olympus microscope (Watford). For each treatment group, three fields were randomly selected for observation, and three biological replicates were performed.

### Real-Time PCR

Neuroblastoma cells (SK-N-AS, SK-N-BE-(2)­C, SK-N-DZ, and SK-N-SH) were seeded in 6-well plates at a density of 4 × 10^5^ cells per well. After 24 h, the cells were treated with 10 μM sirolimus, 5 μM pyrvinium pamoate, or DMSO for 6 h. After treatment, cells were washed twice with cold PBS and lysed with TRIzol Reagent (Invitrogen) and chloroform (Sigma-Aldrich). Total RNA was extracted using the Direct-zol RNA MiniPrep Kit (Zymo Research) following the manufacturer’s instructions. Reverse transcription of 500 ng of RNA to cDNA was performed using the qRT-PCR Kit (Thermo Fisher Scientific). qRT-PCR was then conducted using an iQ SYBR Green Supermix (Bio-Rad Laboratories) on a CFX Connect Real-Time PCR Detection System (Bio-Rad Laboratories).

The primer sequences used are as follows:

Human Cyclin A2 (*CCNA2*): 5′ -CGCTGGCGGTACTGAAGTC-3′ (Forward) and reverse 5′ -GAGGAACGGTGACATGCTCAT-3′ (Reverse); Human Centromere Protein A (*CENPA*): 5′-CTCTGCGGCGTGTCATGG-3′ (Forward) and 5′-GCCGACTGTGTTGATGGGAGG- 3′ (Reverse).

Human Excision Repair Cross-Complementation Group 6 Like (*ERCC6L*): 5′ -CAGTTGGTTGGTTCTCCCCA-3′ (Forward) and 5′ -AGGGCCTCCTGGATTTTTCC-3 ′ (Reverse).

Human Flap Endonuclease 1 (*FEN1*): 5′-AAGTCTATGCTGCGGCTACC-3′ (Forward) and 5′-TGGATTGGCAGCTTTTTGGC-3′ (Reverse).

Human Holliday Junction Recognition Protein (*HJURP*): 5′-CTGCCCAAGAGCGATTCATC-3′ (Forward) and 5′-GTAACGATTCCTTCCGTGGC- 3′ (Reverse).

Human Kinesin Family Member 4A (*KIF4A*): 5′-GTCTGGCTTGGGAGATGCTT-3′ (Forward) and 5′-GGCTAAGGCCCACATCCAAC- 3′ (Reverse).

Human Kinesin Family Member 20A (*KIF20A*): 5′-ACTGCTCTGTCGTCTCTACCT- 3′ (Forward) and 5′-GGTAACAAGGGCCTAACCCTC-3′ (Reverse).

Human Mitotic Arrest Deficient 2 Like 1 (*MAD2L1*): 5′-CGTGCTGCGTCGTTACTTTT-3′ (Forward) and 5′-GCCGAATGAGAAGAACTCGG- 3′ (Reverse).

Human Minichromosome Maintenance Complex Component 3 (*MCM3*): 5′-CTGAAGGCGAGGAATGTTGGTG-3′ (Forward) and 5′-GATGGGAAGTAGGGCGGATGAG-3′ (Reverse).

Human Minichromosome Maintenance Complex Component 10 (*MCM10*): 5′-GCATGATGGTGTGAAGAGGTTT-3′ (Forward) and 5′-TCCCATTTGTAGAGGCCACAG-3′ (Reverse).

Human Nei Like 3 (*NEIL3*): 5′-TGGACATCTAGCAGGGTGGA-3′ (Forward) and 5′-CACACAGGTCCAGTGCTCTT-3′ (Reverse).

Human RuvB Like AAA ATPase 1 (*RUVBL1*): 5′ -GGAGGTGAAGAGCACTACGA-3′ (Forward) and 5′ -ACTATGACGCCACATGCCTC-3′ (Reverse).

Human glyceraldehyde-3-phosphate dehydrogenase (*GAPDH*): 5′ -ACACCCACTCCTCCACCTTTG-3′ (Forward) and 5′ -GCTGTAGCCAAATTCGTTGTCATAC-3′ (Reverse).

Differential expression was determined using CFX Maestro software v2.1 (Bio-Rad Laboratories).

### Sample Preparation for Quantitative Proteome Analysis

PBS-washed cells were scraped in cell lysis buffer containing 12 mM sodium deoxycholate (Sigma-Aldrich), 12 mM sodium *N*-lauroylsarcosinate (Sigma-Aldrich), and 100 mM Tris–HCl (pH 9.0, Sigma-Aldrich), supplemented with a protease inhibitor cocktail (Thermo Fisher Scientific). Lysates were sonicated for 2 min on ice to shear genomic DNA. Protein concentrations were determined by a BCA assay (Thermo Fisher Scientific). Fifty micrograms of proteins was reduced with 10 mM TCEP (Merck) for 30 min, alkylated with 25 mM chloroacetamide (Merck) for 30 min, and digested with 0.5 μg of Lys-C (Wako) for 3 h, followed by 0.5 μg of trypsin (Promega) overnight in 50 mM ammonium bicarbonate (Sigma-Aldrich).

Digestion was terminated by the addition of trifluoroacetic acid (TFA, Sigma-Aldrich) to a final concentration of 0.5%, and detergents were extracted by adding ethyl acetate (Merck, 1:1 v/v). The peptide mixture was then desalted using reversed-phase StageTips^39^ and quantified by Pierce Quantitative Colorimetric Peptide Assay (Thermo Fisher Scientific). An equal amount of desalted peptides from each sample was dried and subjected to isobaric TMT labeling in 200 mM HEPES for 1 h. The reactions were quenched with 0.33% hydroxylamine for 15 min. Multiplexed labeled samples were combined, dried, and fractionated using basic reversed-phase fractionation^40^ on C18 StageTips into 8 fractions. Resulting fractions were desalted using a C18 StageTips.

### Nano LC–MS/MS and Data Analysis

Peptide samples were dissolved in 0.1% formic acid (FA) and analyzed on an Orbitrap Fusion Lumos Tribrid quadrupole-ion trap-Orbitrap mass spectrometer (Thermo Fisher Scientific) equipped with an Ultimate 3000 nanoLC system (Thermo Fisher Scientific). Peptides were loaded into a C18 Acclaim PepMap NanoLC column (25 cm length and 75 μm inner diameter) (Thermo Fisher Scientific) packed with 2 μm particles having a pore of 100 Å. The mobile phase A consisted of 0.1% FA in water, and mobile phase B was 100% acetonitrile (ACN) with 0.1% FA. Samples were separated by gradually increasing the ACN from 2% to 40% containing 0.1% FA in 50 min at a flow rate of 300 nL/min.

The mass spectrometer operated in data-dependent mode, automatically switching between the MS1 and MS2 (MS/MS) acquisition. MS1 spectra ranging from 350 to 1700 *m*/*z* segments were acquired in the Orbitrap at a resolution of 120,000 at 200 *m*/*z* with an AGC target of 5 × 10^5^ and a maximum injection time of 50 ms. The instrument was set to run in top speed mode with 3 s cycles for both the survey and the MS/MS scans. Peptide ions with charge states 2–7 were sequentially selected with an isolation width of 1.4 Da for fragmentation by higher-energy collisional dissociation at normalized collision energy of 38%. The resulting fragment spectra were acquired in the Orbitrap mass analyzer with a resolution of 60,000. An AGC target of 5 × 10^4^ was set for MS/MS analysis with dynamically excluded previously selected ions for 60 s.

Peptides were identified with MaxQuant^41^ software (version 1.6.17.0) against the SwissProt Database (version 2020_02) utilizing the Andromeda peptide search engine.^42^ Reporter ion MS2 was adjusted for 11-plex TMT isobaric labels. Variable modifications included methionine oxidation and N-terminal protein acetylation, while carbamidomethylation of cysteine residues was a fixed modification. Trypsin/P was set as the enzyme with specificity for the digestion mode, allowing a maximum of two missed cleavages. The “matching-between-runs” option was enabled with default parameters. The ProteinGroups output file from MaxQuant was further processed and analyzed with Perseus version 1.6.14.0.^43^

### Principal Component Analysis

To confirm the performance of samples in the proteome analysis, we conducted PCA with “prcomp” function from stats package (version 4.3.1) in the R environment (version 4.3.1).^44^ We input data containing the intensity of each protein from the proteome experiment. The PCA analysis was performed with the default settings. By utilizing principal components 1, 2 (PC2), and 3 (PC3) of the proteome data, we generated a three-dimensional plot to identify within-group similarity and between-group dissimilarity. The visualization was created using the “add_trace” function from plotly package (version 4.10.4).^45^

### Functional Enrichment with Gene Ontology Analysis

To perform functional enrichment analysis using GO, official gene symbols of the differentially expressed proteins (DEPs) with *p*-value <0.05 and fold change < −1.96 standard deviations were analyzed by the Database for Annotation, Visualization, and Integrated Discovery (DAVID) 6.8 (https://david.ncifcrf.gov/)^46^ with specified as the species. The terms from the GO biological process (GOBP) and KEGG_PATHWAY were used for analysis. The enriched terms with a *p*-value of less than 0.05 were considered significant.

#### Gene Set Enrichment Analysis

GSEA was performed using GSEA 4.0.1 software.^47^ The analysis targeted GO terms, and significant enrichment was determined through 1000 permutations. A pathway was considered significantly enriched if it had a false discovery rate value < 0.25 and a normalized *p*-value <0.05.

### Cell Migration Assay

To assess cell migration, we conducted an assay using transwell plates with 8 μm pore size (Corning). We seeded 2 × 10^6^ neuroblastoma cells onto a 10 cm culture dish and allowed them to adhere for 1 day. Then, we treated the cells for 24 h with 1 μM pyrvinium pamoate, 16 μM sirolimus, and 1 μM pyrvinium pamoate plus 16 μM sirolimus, respectively. Next, we seeded 5 × 10^4^ treated neuroblastoma cells in serum-free medium into the upper inserts, while the lower compartments were filled with medium containing 10% FBS. The cells were incubated at 37 °C for 12 h, fixed with 100% methanol for 2 h, and stained with 0.1% crystal violet overnight. Cells remaining on the upper side of the insets were removed with cotton swabs. We captured images of three fields from each insert and counted the number of cells.

### Western Blotting

Neuroblastoma cells were harvested, and proteins were extracted using radioimmunoprecipitation assay buffer (50 mM Tris–HCl (pH 7.4), 150 mM NaCl, 1% (v/v) NP40, 0.5% (w/v) sodium deoxycholate, and 0.1% SDS), supplemented with 10% protease inhibitor cocktail (BioShop) and phosphatase inhibitor cocktail I and II (BioShop). Samples were sonicated using a LABSONIC M ultrasonic (Montreal Biotech Inc.) homogenizer for 2 min. Protein concentration was determined by using a BCA protein assay kit (Thermo Fisher Scientific). Proteins were separated by 12% sodium dodecyl sulfate-polyacrylamide gel electrophoresis and transferred onto polyvinylidene difluoride membranes (Merck Millipore). The membrane was blocked with 5% BSA in tris-buffered saline-tween (TBST) for 1 h at room temperature and incubated with specific primary antibodies overnight at 4 °C. We used the following primary antibodies: MRPL15 (GTX122571, 1:500, GeneTex), UBE2C (GTX100599, 1:500, GeneTex), AKR1C3 (GeneTex, GTX104627, 1:500), GOLGA3 (GTX100288, 1:500, GeneTex), ELP2 (GTX121449, 1:500, GeneTex), LC3B (GTX127375, 1:500, GeneTex), p62 (GTX100685, 1:1000, GeneTex), Caspase3 and cleaved Caspase3 (GTX110543, 1:500, GeneTex), PARP and cleaved PARP (GTX100573, 1:5000, GeneTex), and GAPDH (GAP001R, 1:5000, Bioman) as the internal control. The following day, the membrane was washed three times with TBST for 10 min each to remove excess antibodies and then labeled with a secondary antibody at room temperature for 1 h. The blots were visualized by using enhanced chemiluminescence (Millipore).

### Statistical Analysis

The results were presented as the mean ± standard deviation. Differences between two groups were compared using the Student’s *t*-test. A *p*-value of less than 0.05 was considered statistically significant. All experiments were conducted in triplicate.

## Results

### Combination Treatment of Sirolimus and Pyrvinium Pamoate Decreased the Proliferation of Neuroblastoma

In recent years, combination therapy has emerged as a new avenue for cancer treatment involving the use of multiple drugs. In our previous research, we analyzed experimental data from various cell types exposed to various drugs, focusing on changes in mRNA expression, chemical and genetic perturbagens, and transcriptomic profiles available in the Library of Integrated Network-Based Cellular Signatures (LINCS) to predict and identify combinatorial drugs with potential therapeutic synergy.^11^ The combination of sirolimus and pyrvinium pamoate was identified as a promising candidate for neuroblastoma treatment, with subsequent experiments confirming their effectiveness in inhibiting colony growth in neuroblastoma cell lines. We first assessed the toxic effects of these drugs individually on neuroblastoma cell lines, including SK-N-BE-(2)­C, SK-N-DZ, SK-N-AS, and SK-N-SH. The results showed that as the concentration of sirolimus or pyrvinium pamoate increased, and with longer treatment durations, the survival rate of neuroblastoma cells significantly decreased, indicating that both drugs have inhibitory effects on neuroblastoma growth (Figure 1A,B).

**Figure 1:**
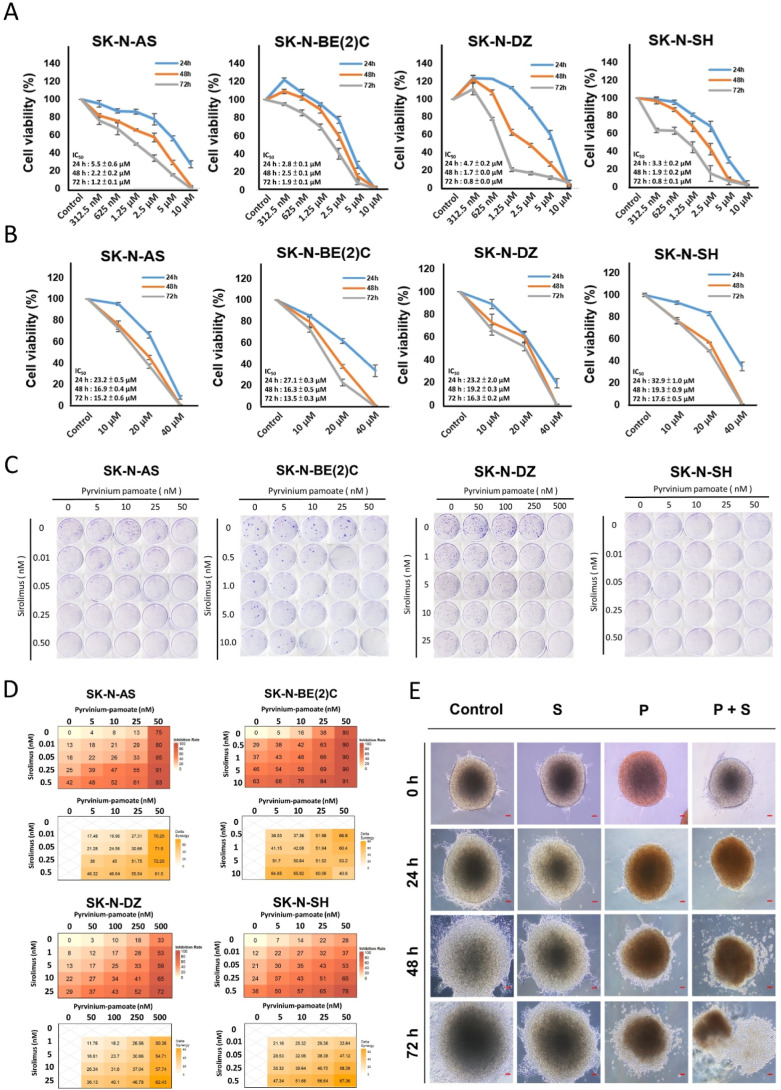
Analysis of cell viability in neuroblastoma cell lines following combination treatment with sirolimus and pyrvinium pamoate. (A,B) Neuroblastoma cells were seeded in a 96-well plate at a density of 5000 cells per well and incubated for 24 h, 48 h, or 72 h. Cells were then treated with varying concentrations of pyrvinium pamoate (0.3125, 0.625, 1.25, 2.5, 5, and 10 μM); panel (A) or sirolimus (0, 10, 20, and 40 μM); panel (B). Cell viability was measured using the CellTiter-Glo Luminescent cell viability assay kit or the MTS assay. (C) Four neuroblastoma cell lines, including SK-N-AS, SK-N-BE(2)­C, SK-N-DZ, and SK-N-SH, were subjected to various concentrations of combined therapy with sirolimus and pyrvinium pamoate over a period of 10 days. (D) Heatmaps showing the inhibition rates (top) and synergy scores (bottom) across various concentration combinations of pyrvinium pamoate and sirolimus. Inhibition rates were quantified from the colony formation assay results shown in panel (C), and synergy scores (delta scores) were calculated based on these data to assess the nature of drug interactions. (E) Organoids with a diameter of 600–650 μm were seeded onto Matrigel-coated 12-well plates and cultured. The organoids were treated with varying concentrations of sirolimus (20 μM) or pyrvinium pamoate (2 μM). Morphological changes were observed at 0, 24, 48, and 72 h post-treatment. Red bar = 70 μμm.

Next, we assessed the long-term efficacy of the combination treatment in neuroblastoma cell lines using colony formation assays (Figure 1C). As a long-term assay, colony formation provides a robust evaluation of the treatment effectiveness and is particularly suitable for testing lower drug concentrations over extended periods. Despite some variability among different cell lines, the combination treatment significantly suppressed colony growth at the optimized concentrations. Notably, a synergistic effect was observed, as the effective concentration required for the combination was substantially lower than that of either drug alone, indicating the potential for enhanced efficacy with reduced toxicity (Figure 1D). To further explore the therapeutic potential of the combination, we extended our analysis to patient-derived organoids (PODs). As shown in Figure 1E, at the beginning of the treatment (24 h), the morphology of the organoids under combined treatment was still a complete spherical shape. In the control group and the two single-drug treatments, the organoids began to expand and grow. At 48 h, the organoids under the combined treatment began to disintegrate and die. The control group and the two single-drug treatments, organoids, still showed an expansion growth pattern. At 72 h, the combined treatment showed large-scale disintegration and a large number of dead cells. The control group and sirolimus treatment still showed an expansion growth pattern, while pyrvinium pamoate treatment showed that a small number of dead cells appeared under drug treatment, but the cells still showed an expanding growth pattern. These findings underscore the potential of a synergistic therapeutic approach using these two agents in neuroblastoma treatment.

### Gene Expression and Pathway Analysis Unveiling the Possible Mechanisms of Combination Treatment of Sirolimus and Pyrvinium Pamoate

Using a drug screening method developed in our laboratory,^11^ an initial drug screening identified 42 genes affected by sirolimus or pyrvinium pamoate. Among these, we selected 12 genes (Figure 2A), Cyclin A2 (*CCNA2*), Minichromosome Maintenance Complex Component 10 (*MCM10*), Excision Repair Cross-Complementation Group 6 Like (*ERCC6L*), Kinesin Family Member 20A (*KIF20A*), RuvB Like AAA ATPase 1 (*RUVBL1*), MAD2L1, Minichromosome Maintenance Complex Component 3 (*MCM3*), Nei Like 3 (*NEIL3*), Kinesin Family Member 4A (*KIF4A*), CENPA, HJURP, and FEN1, which were most closely associated with neuroblastoma survival rates (Figure S1). We then examined the effects of the drugs on mRNA expression levels in neuroblastoma cells. To validate these predictions, we conducted quantitative mRNA analysis on the 12 genes and confirmed that treatment with either pyrvinium pamoate (Figure 2B) or sirolimus (Figure 2C) altered the expression levels of these genes across multiple neuroblastoma cell lines. To further understand the physiological pathways altered by combination therapy that led to the suppression of neuroblastoma cell survival, we subjected these 12 genes to GOBP term enrichment analysis using DAVID and revealed that most GO terms were associated with cell division and DNA replication (Figure 3A and Table S1), supporting the high inhibitory efficacy of the drug combination. Our GO term analysis suggested that neuroblastoma cells may influence cell division and the mitotic process (Figure 3A and Table S1). To validate this finding, we selected treatment concentrations based on mRNA expression levels and performed fluorescence immunostaining to examine the most prominent biological processes, particularly mitotic division, to assess whether the combination therapy affects cell division. Neuroblastoma cell lines were synchronized and treated with either the combination of 10 μM sirolimus and 5 μM pyrvinium pamoate or an equivalent volume of DMSO as a control. Compared to the control group, the treatment group exhibited a reduction in mitotic division, indicating that combination therapy disrupts the mitotic process in neuroblastoma cells (Figures 3B and S2). The results further demonstrated that under dual-drug treatment cells failed to undergo normal mitosis, leading to mitotic catastrophe, suggesting that the combination therapy effectively interferes with cell division.

**Figure 2:**
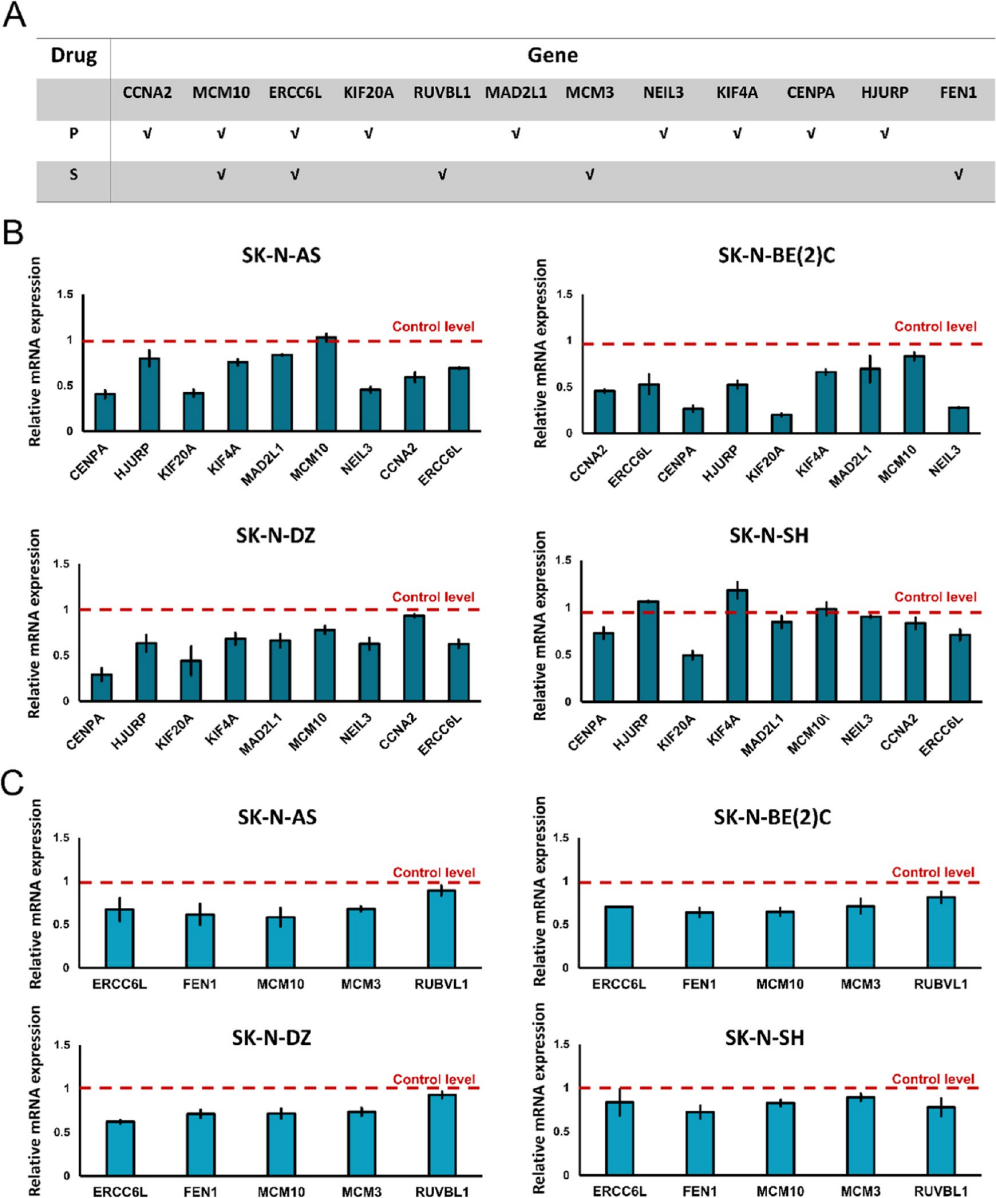
Pyrvinium pamoate and sirolimus decrease mRNA expression levels in neuroblastoma cell lines. (A) Identification of 12 genes associated with neuroblastoma influenced by either sirolimus or pyrvinium pamoate. The extent to which genes affect the survival rate of neuroblastoma decreases from left to right. Checkmark (√) indicates the genes predicted to be affected by each drug during the initial drug screening process. P, pyrvinium pamoate. S, sirolimus. (B,C) 4 × 10^5^ Cells/well were seeded into 6-well plates, incubated for 24 h, and then treated with 0 or 5 μM pyrvinium pamoate (B) or 10 μM sirolimus (C) for 6 h. After treatments, RNA was extracted using TRIzol, and cDNA synthesis was performed. qRT-PCR was conducted using iQ SYBR Green Supermix on a Real-Time PCR Detection System. The dotted line across 1 in the figure represents the expression level of mRNA in the control group.

**Figure 3:**
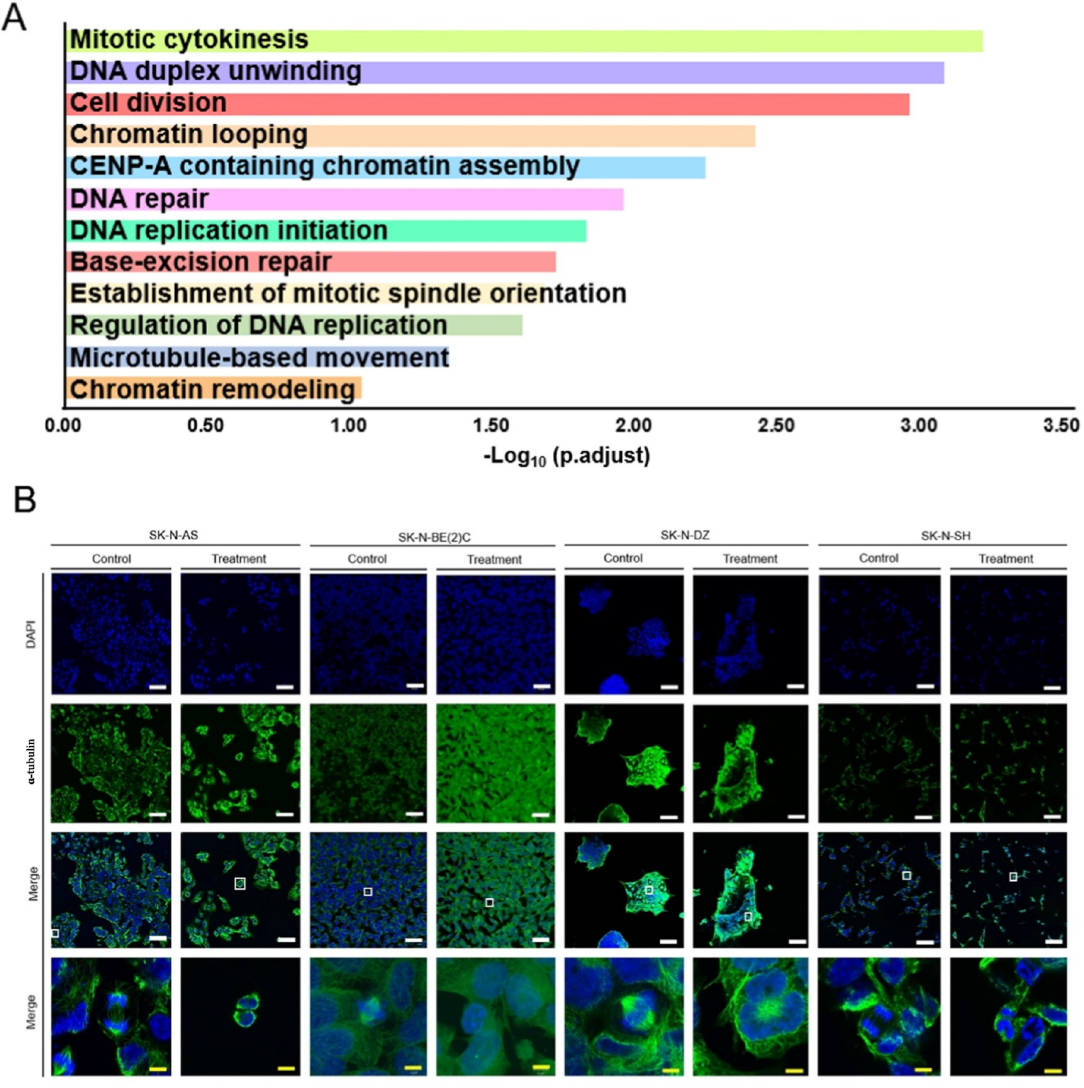
12 genes downregulated by treatment with pyrvinium pamoate and sirolimus inhibit cell mitosis. (A) Functional enrichment analysis was performed by inputting the 12 genes into DAVID 6.8 (https://david.ncifcrf.gov/), focusing on biological processes (BP) for GO term analysis. (B) The effect of combined therapy on mitosis in neuroblastoma cell lines. Cells were treated with 0 or 10 μM sirolimus and 5 μM pyrvinium pamoate for 8 h. Fixed cells were treated with 3.7% paraformaldehyde for 15 min, blocked in PBS with 5% BSA for 1 h, and then incubated with primary antibodies (α-tubulin) overnight at 4 °C. Secondary antibodies were incubated for 1 h. Slides were counterstained with DAPI. White bar = 50 μm. Yellow bar = 5 μm. The region outlined by the white box in the Merge images is shown at 630× magnification in the panel on the right to highlight the detailed features.

### Quantitative Proteomic Analysis Reveals the Impact of Combination Therapy

To validate the actual synergistic effects, we further investigated the impact of combined treatment on the global protein expression by a TMT-assisted LC–MS/MS-based proteomics approach (Figure 4A). In this study, we selected the SK-N-DZ cell line primarily because its mRNA expression profile closely matched our predicted outcomes (Figure 2B,C). Additionally, SK-N-DZ cells exhibit significant MYCN amplification, which was evident from their response in the colony formation assay (Figure 1C), indicating a requirement for higher drug concentrations to achieve effective combination therapy. MYCN amplification is frequently associated with high-risk neuroblastoma cases and poor prognosis, underscoring the importance of focusing on this cell line for subsequent experiments. PCA confirmed the consistency of the treatments (Figure 4B). In total, we identified 3416 protein groups from 20,623 peptides with 2445 quantifiable protein groups (Figure 4C and Table S4). DEPs were defined base on statistically significant differences (*p*-value <0.05) and a biologically meaningful threshold (|log_2_ fold change| > 0.58). Using these criteria, we identified 26, 97, 27, 133, 26, and 239 DEPs in the following comparisons: pyrvinium pamoate vs DMSO, dual drug vs DMSO (batch 1), sirolimus vs DMSO, dual drug vs DMSO (batch 2), dual drug vs pyrvinium pamoate, and dual drug vs sirolimus, respectively (Figure 4D–I). Notably, more DEPs were observed in the comparison between the dual-drug treatment (pyrvinium + sirolimus) and sirolimus alone (Figure 4I) than between the dual-drug treatment and pyrvinium alone (Figure 4H), suggesting that 1 μM pyrvinium pamoate exerts a dominant effect in the combination treatment. The details of DEPs for each treatment group are given in Tables S2 and S3. To validate the proteomics results, we randomly selected several DEPs and confirmed the expression levels by Western blot analysis (Figure S3).

**Figure 4:**
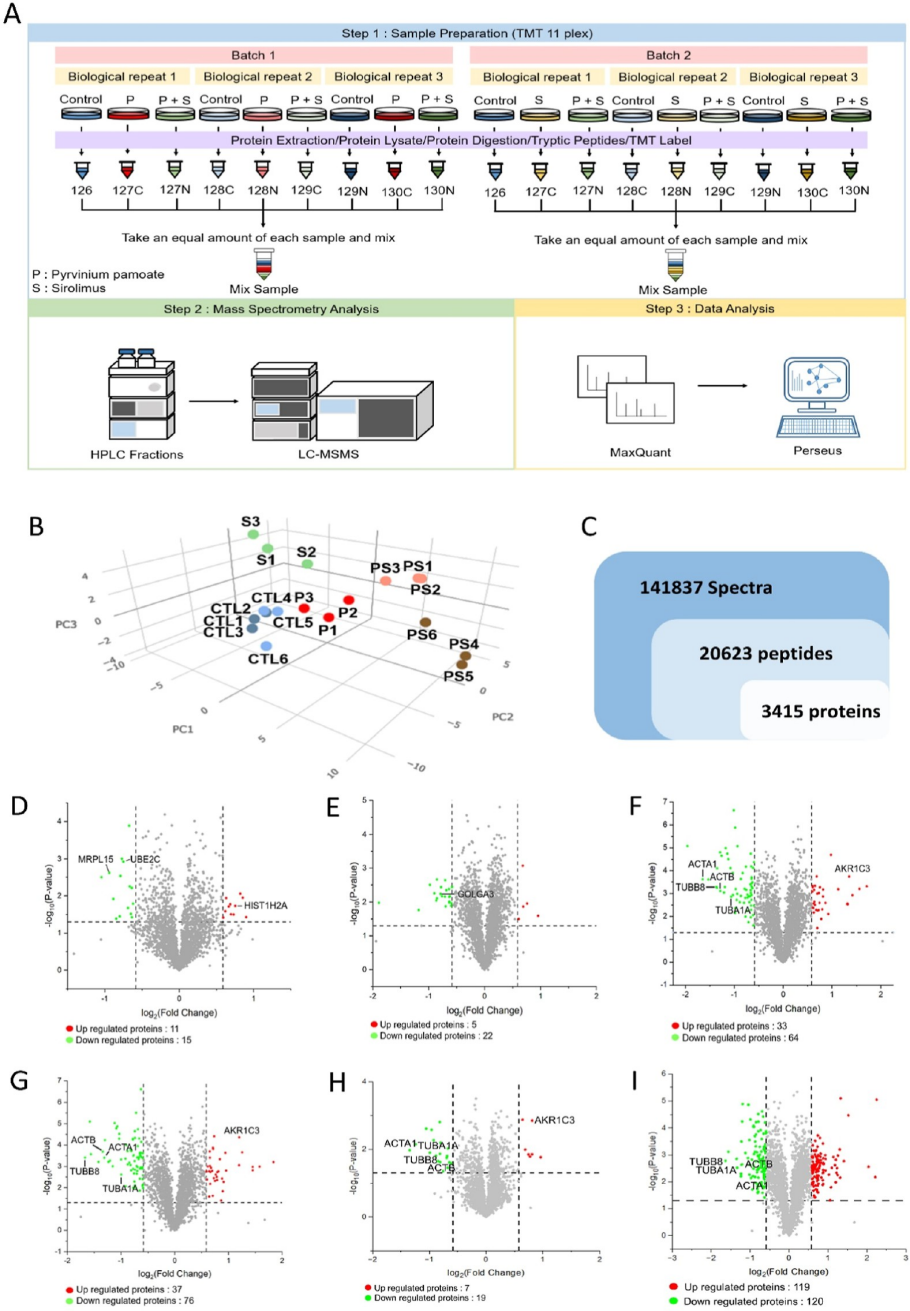
Quantitative proteomics of treatment with pyrvinium pamoate, sirolimus, or combination therapy in SK-N-DZ. (A) Mass spectrometry experimental design. We employed the 11-plex TMT system for sample labeling. To elucidate the differences in intracellular protein expression between monotherapy and combination therapy, we divided the samples into two batches. After preprocessing, the samples underwent mass spectrometry analysis. Subsequently, MaxQuant and Perseus were utilized for protein identification, quantification, and statistical analysis. (B) Principal components analysis presentation of all samples in this research. CTL: control, P: pyrvinium pamoate, S: sirolimus, and PS: pyrvinium pamoate + sirolimus. (C) Quantitative Proteome Profiling: Our quantitative proteomics analysis identified 141,837 spectra, corresponding to 20,623 peptides and 3415 proteins. (D–I) To reveal the expression differences of proteins under different experimental conditions, we used a volcano plot to visualize and analyze the quantitative proteomics data with the *x*-axis representing the log2 fold change and the *y*-axis representing the -log10 *p*-value. We analyzed different experimental conditions as follows: (D) P/C, (E) S/C, and (F) PS/C in batch 1; (G) PS/C in batch 2; (H) PS/P; and (I) PS/S.

### Functional Enrichment Analysis Revealed the Molecular Mechanisms of Dual-Drug Treatment

We subsequently examined the effects of the drug on proteome alterations using the DAVID and GSEA, with a focus on biological processes (Figure 5A). Specifically, we focused on the downregulated functions. The downregulated DEPs (Table S3) from each comparison group were subjected to DAVID. In contrast, we imported the whole expression data into GSEA and focused on terms exhibiting negative enrichment scores (NES <0). This approach enabled us to distinguish which mechanisms were inhibited by the combined drug treatment. Under single-drug conditions, pyrvinium pamoate primarily exhibited mitochondrial-associated changes (Figures 5B and S4A and Table S5, S6), while sirolimus treatment affected pathways associated with splicing, as well as the polarity and extension of the cellular cytoskeleton proteins (Figures 5C and S4B and Table S7, S8).

**Figure 5:**
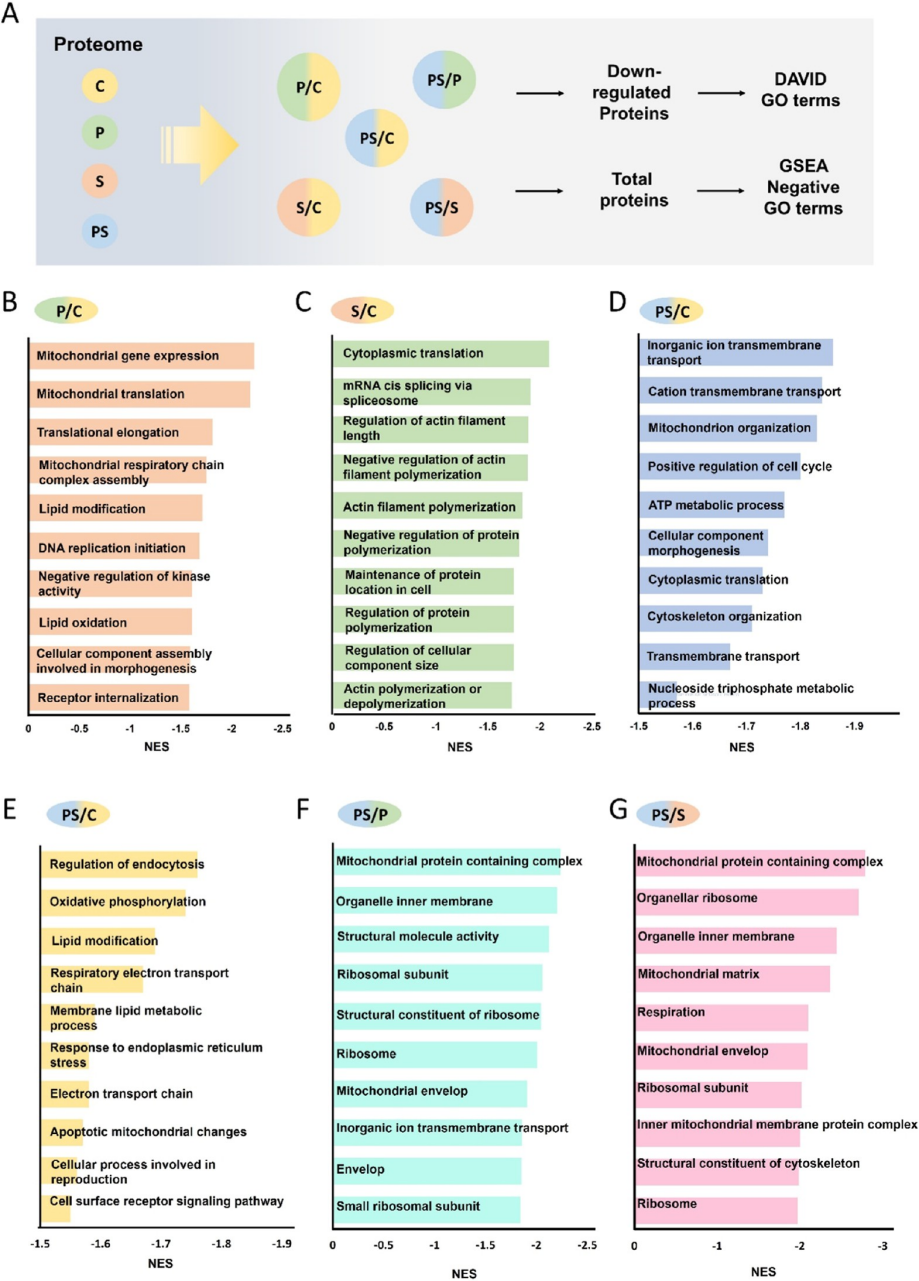
The biological process analysis of monotreatment and combined treatment. (A) GSEA identified significant differential GO terms by comparing the proteomic data (fold changes) between different groups. C: control, P: pyrvinium pamoate, S: sirolimus, and PS: pyrvinium pamoate + sirolimus. (B–G) Key terms identified through GSEA illustrate the differences in the effects of monotreatment (P/C and S/C) and combined therapy compared to control group (PS/C). Additionally, the influence of combined therapy compared to individual treatments (PS/P or PS/S) are also illustrated.

Furthermore, when comparing the proteomic alterations under dual-drug treatment to the control group, we consistently observed mitochondria-related changes in both DAVID and GSEA analyses (Figures 5D,E and S4C,D and Tables S9–S12), indicating minimal disparities between dual-drug treatment and pyrvinium pamoate monotherapy (Figures 5B and S4A). Additionally, we identified significant enrichment of cell cycle-related DEPs under dual-drug treatment, which aligns with the key gene analysis results predicted in the previous study. This demonstrates the consistency between drug analysis and actual proteomic experiments, highlighting the pivotal role of cell cycle downregulation in this context (Figures 3B and S2).

We found that the effects of the dual-drug treatment closely resemble those of pyrvinium pamoate, as highlighted by the enrichment of functions related to mitochondrial metabolism (Figures 5F,G and S4E,F and Tables S13–S16). Nevertheless, while the influence on the cytoskeleton was enriched in sirolimus monotherapy (Figures 5C and S4B), the impact on cytoskeletal organization was significantly more pronounced in the dual-drug treatment compared to that of either monotherapy alone (Figure 5G). Given that the cytoskeleton is crucial for cell migration, we suggested it might significantly impact the migration behavior of neuroblastoma.

### Combination Therapy Induced Cell Migration and Cell Death through Autophagy

To further confirm whether cell migration is affected by dual-drug treatment, we conducted immunofluorescence staining to inspect the effects of the treatment on cellular cytoskeletal dynamics (Figure 6A). We further performed migration assay with the same number of cells after drug treatment and found that combined treatment significantly reduced cell migratory activity compared to monotherapy (Figures 6B and S5). Additionally, we observed that the arrangement of cellular cytoskeletal proteins appeared sparser under the combined treatment (Figure 6A), indicating a substantial disruption in cytoskeletal composition. These results demonstrate that dual-drug treatment not only reduces cell proliferation in neuroblastoma but also decreases the migration ability of neuroblastoma cells.

**Figure 6:**
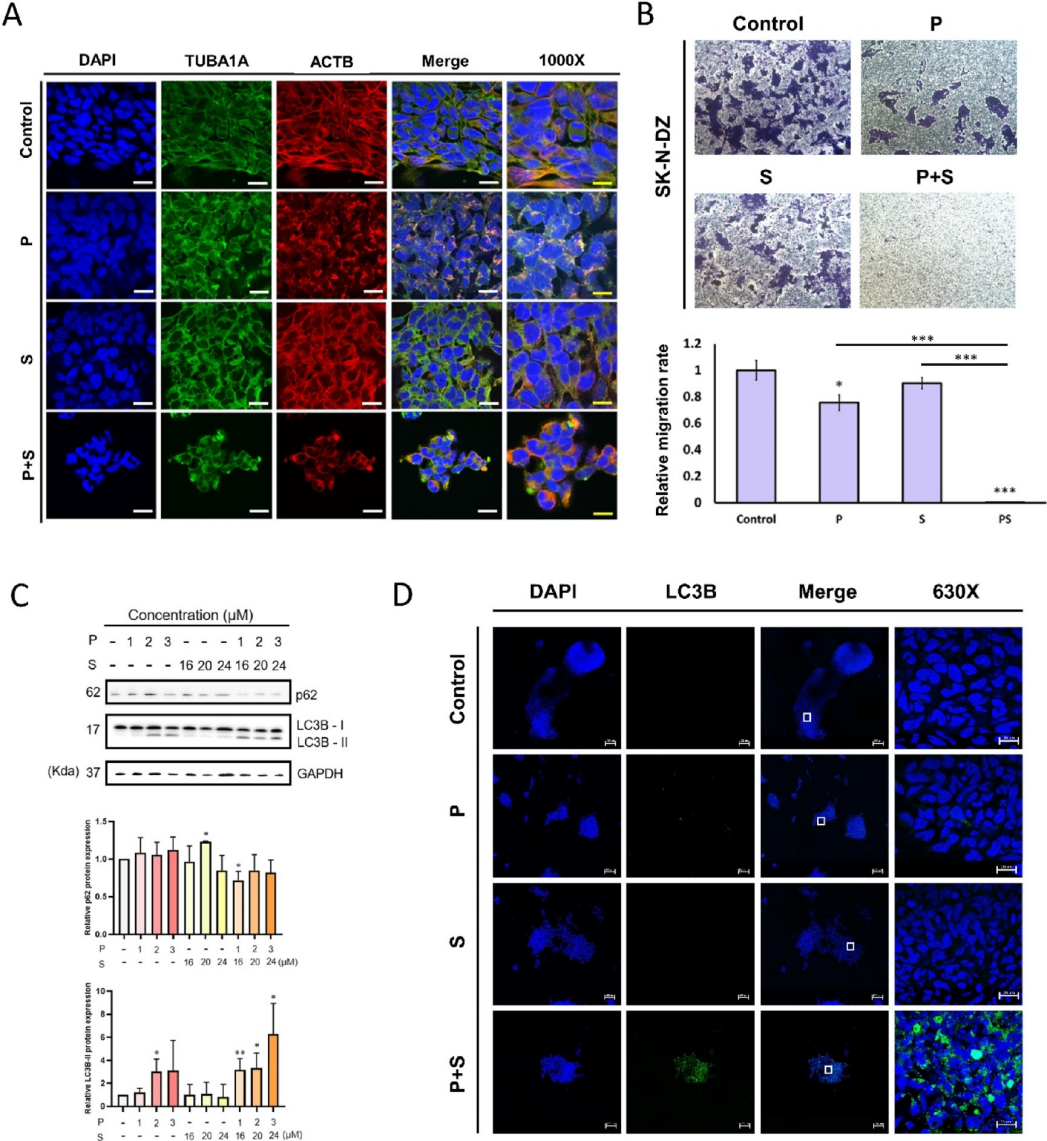
Combination therapy induces cytoskeletal alterations, mitotic catastrophe, and cell death via the autophagy pathway. (A) Conditioned SK-N-DZ cells were fixed in 3.7% paraformaldehyde and incubated with primary antibodies Actin beta (ACTB) or Alpha Tubulin 1A (TUBA1A). The samples were then incubated with secondary antibodies. Slides were counterstained with DAPI and examined under a microscope. White bar = 15 μm. Yellow bar = 10 μm. (B) Conditional SK-N-DZ cells were seeded in 8 μm pore size transwell plates. We seeded 2 × 10^6^ neuroblastoma cells onto a 10 cm culture dish and allowed them to adhere for 1 day. Then, we treated the cells for 24 h with 1 μM pyrvinium pamoate, 16 μM sirolimus, and 1 μM pyrvinium pamoate plus 16 μM sirolimus, respectively. Next, we seeded 5 × 10^4^ treated neuroblastoma cells in a serum-free medium into the upper inserts, while the lower compartments were filled with a medium containing 10% FBS. The cells were incubated at 37 °C for 12 h, fixed with 100% methanol for 2 h, and stained with 0.1% crystal violet overnight. Cells remaining on the upper side of the insets were removed with cotton swabs. We captured images of three fields from each inset and counted the number of cells. (C) The expression levels of autophagy-related proteins were assessed by immunoblotting. (D) Immunocytochemical staining of autophagosome marker LC3B was performed to confirm the autophagy under monotreatment and combination treatment conditions. The region outlined by the white box in the Merge images is shown at 630× magnification in the panel on the right to highlight the detailed features. Blue: DAPI; Green: LC3B; P: pyrvinium pamoate; S: sirolimus; and P + S: pyrvinium pamoate + sirolimus.

Previous studies have highlighted that mitotic catastrophe, if not repaired by the cells, leads to cell death.^48^ This process can occur through autophagy or apoptosis.^49^ Our study indicates that apoptosis is not the primary mechanism of cell death in neuroblastoma cells following dual-drug treatment (Figure S6). The results show that apoptosis was not detected under any of the treatment conditions, suggesting that the observed reduction in cell viability is not due to apoptotic cell death. Instead, our data support the conclusion that autophagy plays a central role in mediating cell death induced by combination therapy. Instead, our findings indicate that autophagy plays a key role in mediating cell death under dual-drug treatment. To validate the impact of the combination treatment on autophagy, we used the same drug concentrations as those in the mass spectrometry experiments and examined the autophagy markers LC3B and p62. During autophagy, LC3B is processed from LC3B-I to LC3B-II through Atg4-mediated cleavage and conjugation to phosphatidylethanolamine, enabling its integration into autophagosomal membranes. p62 serves as a selective autophagy receptor that binds ubiquitinated proteins and LC3, facilitating their delivery to autophagosomes for degradation. Effective autophagy is indicated by LC3B-II accumulation and the concurrent degradation of p62. Western blot analysis revealed that dual-drug treatment led to a substantial increase in LC3B-II levels (more than 2-fold) and a marked reduction in p62 compared to the control group, indicating enhanced and functional autophagy. In contrast, treatment with pyrvinium pamoate alone at 2 or 3 μM caused LC3B-II accumulation without a corresponding decrease in the level of p62, suggesting that autophagy was blocked. Sirolimus alone did not induce detectable autophagic activity at any concentration tested (Figure 6C). These results indicate that the observed autophagy in the combination treatment arises from a synergistic rather than additive effect. Furthermore, immunofluorescence staining demonstrated a significant increase in LC3B levels following dual-drug treatment (Figure 6D), supporting the conclusion that autophagy-dependent cell death may be a key mechanism underlying the enhanced cytotoxicity of the combination therapy.

## Discussion

Given the unique therapeutic alternatives for neuroblastoma patients compared with conventional treatment approaches, this research underscores the potential of combination therapy and reinforces the feasibility of repurposing existing drugs. Unlike many cancers, neuroblastoma lacks well-defined pharmacological targets.^50^ By leveraging the impact of small-molecule drugs on the neuroblastoma transcriptome, we identified compounds with the potential for combination therapy. Utilizing data from LINCS (The LINCS), which includes various cell types, chemical and genetic perturbations, and transcriptomes, we predict several dozen compounds with therapeutic potential. These compounds, whether used individually or in combination, hold promise for neuroblastoma treatment. Among these, some are investigational drugs (not yet approved) (https://www.cancer.gov/about-cancer/treatment/drugs), which did not meet our selection criteria. Others are clinically approved chemotherapeutic agents that, despite their efficacy, come with significant side effects. This is particularly concerning for neuroblastoma, as it predominantly affects children under 10 years old, who could suffer long-lasting physiological and psychological trauma.^51^ Furthermore, the five-year survival rate for high-risk patients undergoing these treatments is approximately 50%,^52^ making these chemotherapy drugs less desirable. Ultimately, we selected sirolimus and pyrvinium pamoate for further experimentation.

Sirolimus and pyrvinium pamoate were approved by the FDA for commercial use in 1999 and 1950, respectively. Sirolimus, derived from ,^53^ was initially developed as an immunosuppressant to reduce organ transplant rejection.^54^ Recently, it has also been approval for treating Lymphangioleiomyomatosis^55^ and is classified as an mTOR inhibitor.^56^ Pyrvinium pamoate, a derivative of quinolone, was originally used as an anthelmintic agent.^57^ It works by inhibiting the mitochondrial electron transport chain^58^ and serves as an inhibitor of the WNT classical pathway.^59^ These mechanisms make both drugs effective targets for cancer treatment.^60^ Notably, both drugs have been on the market for over two decades, indicating their relative safety and widespread availability (https://www.fda.gov/drugs/postmarket-drug-safety-information-patients-and-providers/index-drug-specific-information). Additionally, they are associated with fewer side effects when used within safe dosages compared with traditional chemotherapy agents, which can significantly enhance the quality of life for patients. Therefore, these drugs are considered viable options for cancer patients.^61^ Prior research indicates that both sirolimus^62^ and pyrvinium pamoate^63^ inhibit growth when administered individually. Our study corroborates these findings, demonstrating the ability of each compound to inhibit cell growth in neuroblastoma cells (Figure 1A,B). Interestingly, while prior studies highlighted pyrvinium pamoate’s cytotoxicity during glucose starvation,^64^ our results demonstrate its efficacy against neuroblastoma cells in the absence of glucose starvation (Figure 1B).

In the colony formation assay, we evaluated the effect of prolonged combination therapy on the growth of four neuroblastoma cell lines. The results indicated significant growth suppression across all four cell lines with combination therapy (Figure 1C)., and the same results were also obtained in PODs (Figure 1D). Notably, the effective concentrations for the combination therapy were significantly lower than those required for monotherapy (Figure 1C). Furthermore, combination therapy exhibited pronounced synergistic effects, likely due to interaction of pyrvinium pamoate’s impact on the WNT classic pathway^65^ and sirolimus’s effect on the mTOR pathway.^66^ The mTOR is crucial for cell growth and survival, making mTOR inhibitors a key component of cancer treatment strategies.^67^ Previous research has shown that sirolimus inhibits mTOR, causing cells to arrest in the G1 to S phase transition.^68^ However, mTOR has multiple compensatory mechanisms that promote cell survival and growth.^69^ As a result, sirolimus inhibits tumor growth but does not completely eradicate tumor cells, preventing them from proliferating indefinitely. Most studies have found that sirolimus alone is not as effective as anticipated in cancer treatment. However, coadministration of sirolimus with chemotherapeutic agents can reduce the required dosage of chemotherapy drugs while minimizing adverse effects.^70^ The Wnt pathway is closely associated with cell proliferation and differentiation,^71^ and its aberrant activation has been observed in numerous cancers,^72–74^ making it a significant target in cancer therapy. Studies have indicated that pyrvinium pamoate further inhibits cancer cell growth by suppressing Wnt transduction.^75^ As an upstream pathway to mTOR, WNT jointly and significantly affects cell growth and survival, both influencing growth and survival.^76^ Additionally, pyrvinium pamoate can inhibit the mitochondrial electron transport chain, affecting cellular energy metabolism and ultimately leading to cancer cell death.^77^ The combination therapy demonstrated a significantly more pronounced effect compared to monotherapy, likely due to the interplay of these mechanisms.

Given that many neuroblastoma patients lack specific genetic mutations,^78^ we considered nononcogenic genes associated with neuroblastoma prognosis. This consideration arises from the recognition that single-gene mutations do not reliably predict cell sensitivity to drugs.^79^ Through our screening model, we identified 12 nononcogenic genes (Figure 2A) associated with neuroblastoma prognosis and influenced by either sirolimus or pyrvinium pamoate. Further mRNA expression analysis yielded results consistent with our predictions (Figure 2B, C). These genes are linked to cell cycle regulation, DNA replication, and mitosis. The identified genes participate in various biological functions, with genes such as *CCNA2*, *MCM10*, and *ERCC6L* ^80–82^ playing critical roles in cell division and proliferation. Our analysis indicates a significant downregulation in the expression of these genes post-treatment, suggesting that sirolimus and pyrvinium pamoate may disrupt the cell cycle through the inhibition of these genes in combination therapy. Additionally, genes like *KIF20A*, *RUVBL1*, and MAD2L1,^83–85^ crucial for accurate segregation and alignment during cell division, show a significant decrease in expression levels after drug treatment, supporting the hypothesis of a synergistic effect of these two drugs in combination therapy.

Conversely, genes such as *NEIL3* and FEN1,^86,87^ vital for maintaining genomic stability, exhibit decreased expression levels, indicating a potential reduced ability to respond to DNA damage. This suggests that the combination of sirolimus and pyrvinium pamoate may not only interfere with cell division but also affect cellular DNA repair mechanisms, thereby enhancing the cytotoxicity against neuroblastoma cells. Finally, the validation of genes *CENPA* and *HJURP*,^88,89^ pivotal for chromosome structure and stability, shows a significant decrease in expression postcombination therapy, further confirming the potential synergistic inhibition of neuroblastoma growth through multiple pathways. To determine the cellular physiological pathways affected by these genes, we conducted a GO analysis. This analysis elucidated the physiological impacts of these genes within cells and revealed that these 12 genes are involved in cell division and DNA replication (Figure 3A); these findings are consistent with previous research. Notably, both *ERCC6L* and *MCM10* are influenced by pyrvinium pamoate and sirolimus. These two genes are associated with cell division and DNA replication processes,^90,91^ indicating that combination therapy has greater therapeutic potential for neuroblastoma compared to monotherapy.

DAVID and GSEA analyses indicated diverse biological processes were affected by both treatments. Pyrvinium pamoate primarily influenced mitochondria-related pathways, while sirolimus affects protein synthesis, consistent with prior research.^92,93^ Pyrvinium pamoate also affects lipid metabolism, while sirolimus influenced substance transport, consistent with previous studies.^94,95^ Interestingly, we observed significant differences in up- and downregulated proteins between single-drug and dual-drug treatments. This discrepancy may be due to the increased level of cell damage caused by the combined therapy. Consequently, the effects of the single-drug treatments are amplified under dual-drug conditions, further enhancing the cytotoxic effect. Additionally, we noticed a significant downregulation of structural proteins, including *ACTB* and *TUBA1A*, which are crucial for cell cytoskeleton formation and cell migration.^96,97^ These findings align with our research findings. Immunofluorescence staining confirmed that the downregulation of these genes disrupts cell division processes (Figures 3B and S2), consistent with prior research findings.^98,99^ These results validate our drug screening and prediction models, as the effects of these drugs on the expression of these genes have not been previously investigated.

Finally, in our investigation of cell death mechanisms, our results showed that autophagy plays a crucial role in cell survival and death. Although sirolimus has been previously identified as an inducer of autophagy,^100^ our study did not observe a significant increase in autophagy with sirolimus alone. Similarly, pyrvinium pamoate, known for its autophagy-inhibitory properties,^101^ did not significantly suppress autophagy. These discrepancies may be attributed to differences in cell line differences and variations in the treatment concentrations. In addition, autophagy is a critical pathway leading to cell death. Our results suggest that cell death in our combined treatment was primarily achieved through autophagy. Recent studies have indicated that animals with defective autophagy are more prone to tumor formation,^102^ highlighting the significance of autophagy as an effective pathway for inhibiting tumor survival.

In summary, our study underscores the potential of combination therapy using FDA-approved drugs sirolimus and pyrvinium pamoate for treating neuroblastoma. Through in vitro experiments, we confirmed their therapeutic efficacy and identified potential mechanisms, including the inhibition of cell proliferation, effects on mitochondrial and cellular cytoskeletal metabolism, and the promotion of autophagy. These findings suggest that the combination of sirolimus and pyrvinium pamoate could provide a novel, low-side-effect treatment strategy for patients, offering hope for improved clinical outcomes.

## Supplementary Material





## Data Availability

The original mass spectrometry data has been archived in the jPOSTrepo under Project Accession JPST003261/PXD054625. Complete, uncropped Western blot data are provided in Supplemental Figure S7.

## Notes

Predicted impact of combined therapy in neuroblastoma cell lines; upregulated proteins for quantitative proteomics of treatment with pyrvinium pamoate, sirolimus, or combination therapy in SK-N-DZ; downregulated proteins for quantitative proteomics of treatment with pyrvinium pamoate, sirolimus, or combination therapy in SK-N-DZ; global proteome profile of control, pyrvinium pamoate, sirolimus, and combined treatment for neuroblastoma; DAVID analysis revealed significant differences between pyrvinium pamoate-treated and control-treated SK-N-DZ cells; GSEA revealed significant differences between pyrvinium pamoate-treated and control-treated SK-N-DZ cells; DAVID analysis revealed significant differences between sirolimus-treated and control-treated SK-N-DZ cells; GSEA revealed significant differences between sirolimus-treated and control-treated SK-N-DZ cells; DAVID analysis revealed significant differences between combine-treated and control-treated SK-N-DZ cells from batch 1; DAVID analysis revealed significant differences between combine-treated and control-treated SK-N-DZ cells from batch 2; GSEA revealed significant differences between combine-treated and control-treated SK-N-DZ cells from batch 1; GSEA revealed significant differences between combine-treated and control-treated SK-N-DZ cells from batch 2; DAVID analysis revealed significant differences between combine-treated and pyrvinium pamoate-treated SK-N-DZ cells; DAVID analysis revealed significant differences between combine-treated and sirolimus-treated SK-N-DZ cells; GSEA revealed significant differences between combine-treated and pyrvinium pamoate-treated SK-N-DZ cells; and GSEA revealed significant differences between combine-treated and sirolimus-treated SK-N-DZ cells (XLSX)
Relationship between the expression levels of prognostic genes and overall survival in 498 neuroblastoma patients; whole images and statistics of cell mitosis ratio; validation of proteome analysis; DAVID analysis reveals the distinct impact of pyrvinium pamoate, sirolimus, and their combined treatment on the SK-N-DZ cell line; cell migration inhibited by combined treatment in SK-N-AS, SK-N-BE(2)­C, and SK-N-SH; expression levels of apoptosis-related proteins in each treatment in SK-N-DZ cells; and western blot original images (PDF)
