# Supplementary material for: Quantitative Proteomics Unveils the Synergistic Effects of Combination Drugs on Cytoskeleton Composition and Autophagy-Mediated Cell Death in Neuroblastoma
Source: J Proteome Res. 2025 Jun 17;24(7):3578–96. doi: 10.1021/acs.jproteome.5c00191 (PMC12235702; doi:10.1021/acs.jproteome.5c00191)
Supplement: Supplementary file 2 [file pr5c00191_si_002.pdf]

Supporting Information:

Quantitative Proteomics Unveils Synergistic Effects of Combination drugs on Cytoskeleton Composition and Autophagy-Mediated Cell Death in Neuroblastoma

Pei-Chen Yu[1], Yi-Chun Kao[2], Hsin-Yi Chang[3], Chen-Hao Huang[4], Wen-Ming Hsu[5], Hsuan-Cheng Huang[6,*], Hsueh-Fen Juan[1,2,4,7,*]

[1]Institute of Molecular and Cellular Biology, National Taiwan University, Taipei 106, Taiwan.
[2]Department of Life Science, National Taiwan University, Taipei 106, Taiwan
[3]Graduate Institute of Medical Sciences, National Defense Medical Center, Taipei 114, Taiwan.
[4]Graduate Institute of Biomedical Electronics and Bioinformatics, National Taiwan University, Taipei 106, Taiwan.
[5]Department of Surgery, National Taiwan University Hospital and National Taiwan University College of Medicine, Taipei, Taiwan
[6]Institute of Biomedical Informatics, National Yang Ming Chiao Tung University, Taipei 112, Taiwan
[7]Center for Computational and Systems Biology, National Taiwan University, Taipei 106, Taiwan.

[*]Corresponding Authors
Hsuan-Cheng Huang, Ph.D.
Institute of Biomedical Informatics, National Yang Ming Chiao Tung University, No.155, Sec.2, Linong Street, Taipei 11221, Taiwan; https://orcid.org/0000-0002-3386-0934; Tel: +886-2-28267357; Fax: +886-2-28202508; E-mail: hsuancheng@nycu.edu.tw

Hsueh-Fen Juan, Ph.D.
Department of Life Science, Graduate Institute of Biomedical Electronics and Bioinformatics, National Taiwan University, No. 1, Sec. 4, Roosevelt Rd., 106 Taipei, Taiwan; https://orcid.org/0000-0003-4876-3309; Tel: +886-2-3366-4536; Fax: +886-2-23673374; E-mail: yukijuan@ntu.edu.tw

The Supplementary Information contains two sections:

I. Supplementary Figures (Microsoft Word files)

Fig. S1. The relationship between the expression levels of prognostic genes and overall survival in 498 neuroblastoma patients.

Fig. S2. Whole images and statistics of cell mitosis ratio. The red arrow represents the cell undergoing mitosis.

Fig. S3. Validation of proteome analysis.

Fig. S4. DAVID analysis reveals the distinct impact of pyrvinium pamoate, sirolimus, and their combined treatment on the SK-N-DZ cell line.

Fig. S5. Cell migration inhibited by combined treatment in SK-N-AS, SK-N-BE(2)C and SK-N-SH.

Fig. S6. Expression levels of apoptosis-related proteins in each treatment in SK-N-DZ cells.

Fig. S7. Western blot original images.

II. Supplementary Tables (separate Microsoft Excel files)

Table S1: Predicted impact of combined therapy in neuroblastoma cell lines.

Table S2: Up regulated proteins for quantitative proteomics of treatment with pyrvinium pamoate, sirolimus or combination therapy in SK-N-DZ.

Table S3: Down regulated proteins for quantitative proteomics of treatment with pyrvinium pamoate, sirolimus or combination therapy in SK-N-DZ.

Table S4: Global proteome profile of control, pyrvinium pamoate, sirolimus and combined treatment for neuroblastoma.

Table S5: The DAVID analysis revealed significant differences between pyrvinium pamoatetreated and control-treated SK-N-DZ cells.

Table S6: The GSEA revealed significant differences between pyrvinium pamoate-treated andcontrol-treated SK-N-DZ cells.

Table S7: The DAVID analysis revealed significant differences between sirolimus-treated andcontrol-treated SK-N-DZ cells.

Table S8: The GSEA revealed significant differences between sirolimus-treated and control-treatedSK-N-DZ cells.

Table S9: The DAVID analysis revealed significant differences between combine-treated andcontrol-treated SK-N-DZ cells from batch1.

Table S10: The DAVID analysis revealed significant differences between combine-treated and control-treated SK-N-DZ cells from batch 2.

Table S11: The GSEA revealed significant differences between combine-treated and control-treated SK-N-DZ cells from batch 1.

Table S12: The GSEA revealed significant differences between combine-treated and control-treated SK-N-DZ cells from batch 2.

Table S13: The DAVID analysis revealed significant differences between combine-treated and pyrvinium pamoate-treated SK-N-DZ cells.

Table S14: The DAVID analysis revealed significant differences between combine-treated and sirolimus-treated SK-N-DZ cells.

Table S15: The GSEA revealed significant differences between combine-treated and pyrvinium pamoate-treated SK-N-DZ cells.

Table S16: The GSEA revealed significant differences between combine-treated and sirolimus-treated SK-N-DZ cells.

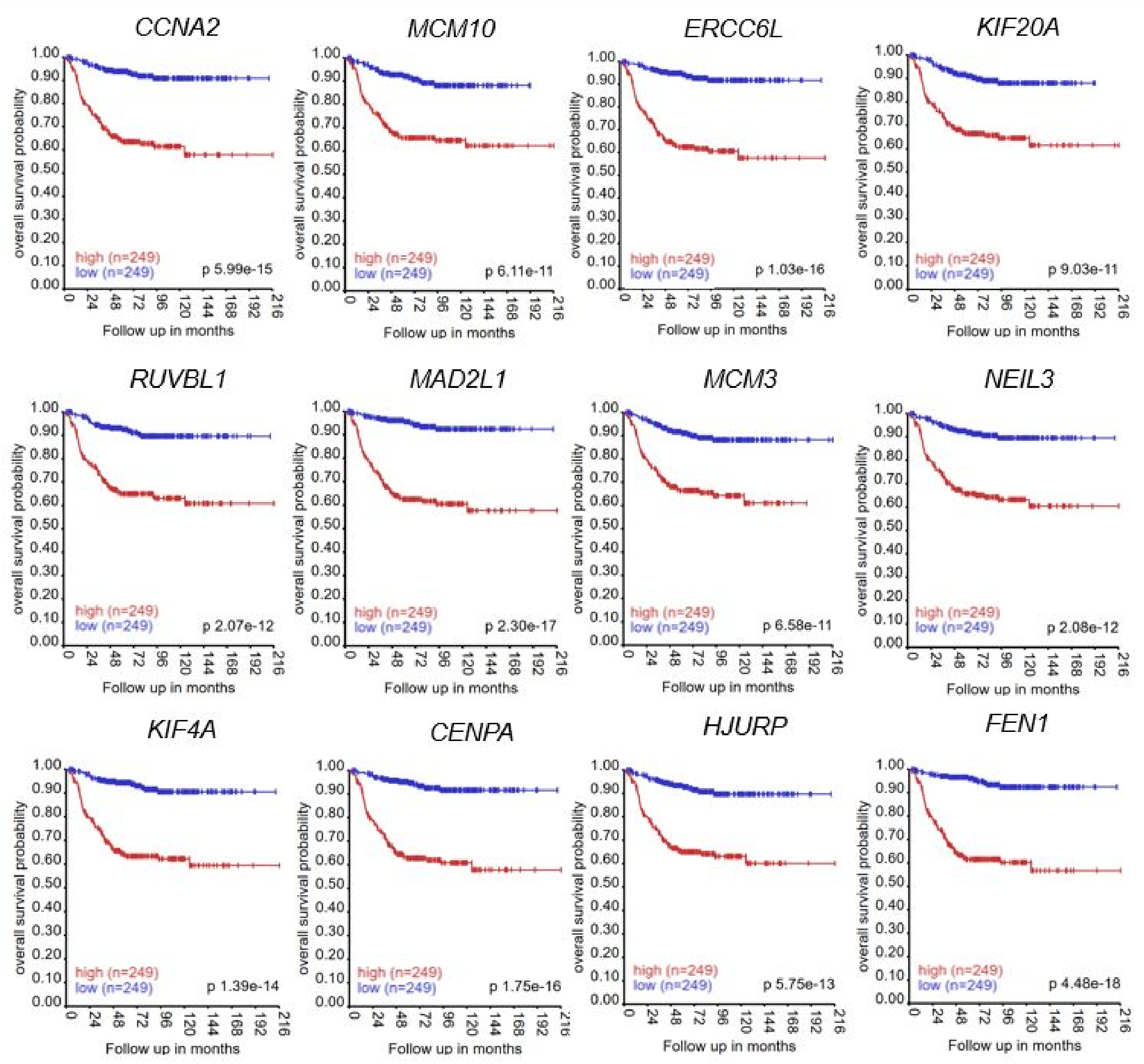

Fig. S1. The relationship between the expression levels of prognostic genes and overall survival in 498 neuroblastoma patients. We utilized the publicly available dataset from GEO (accession number: GSE49710, Agilent-020382 Human Custom Microarray 44k; n = 498) and performed Kaplan-Meier analysis of overall survival for 498 patients using the R2 Genomics Analysis and Visualization Platform, with the median set as the cutoff mode

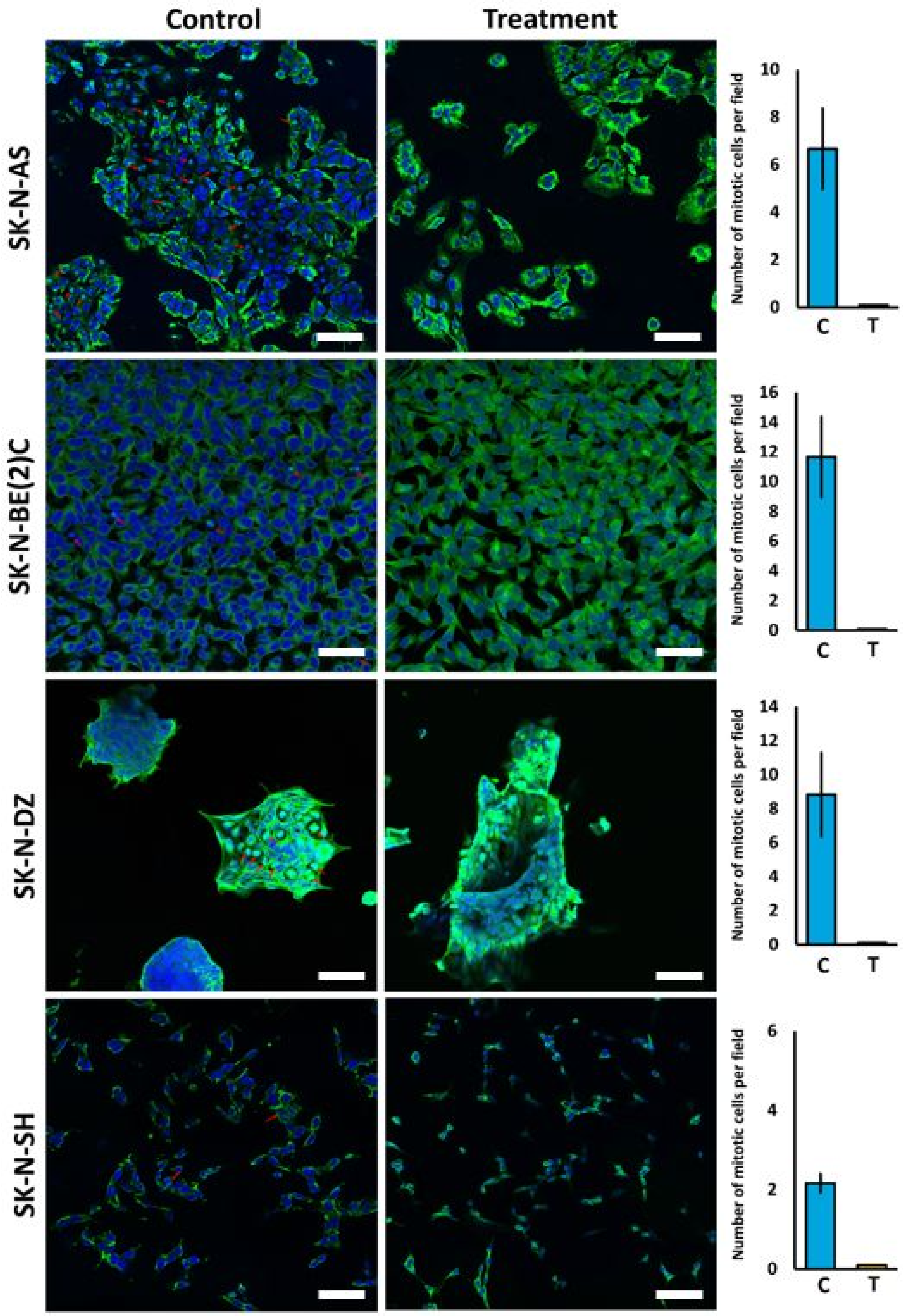
Control
Treatment
SK-N-AS
SK-N-BE(2)C
SK-N-DZ
SK-N-SH
Number of mitotic cells per field
10
8
6
4
2
0
16
14
12
C
T

Fig. S2. Whole images and statistics of cell mitosis ratio. The red arrow represents the cell undergoing mitosis. The image contains nine small fields in each photo. For each treatment group, two large fields were randomly selected for observation (each large field contains nine smaller fields), and three biological replicates were performed. Bar = 50 μm.

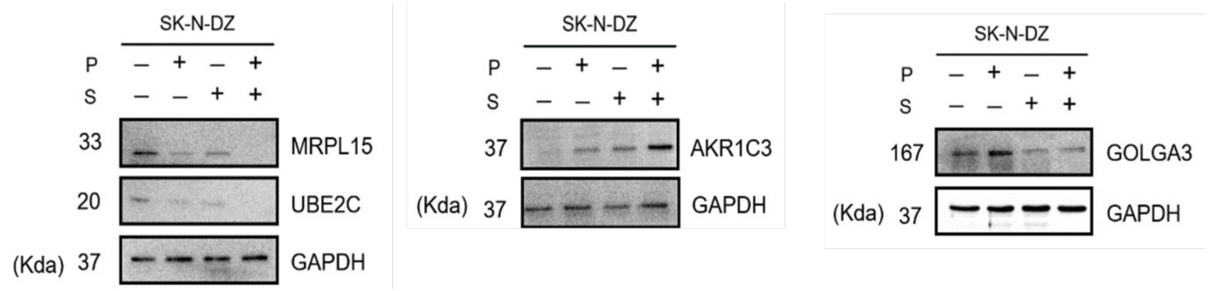

Fig. S3. Validation of proteome analysis. MRPL15 and UBE2C both decrease after pyrvinium pamoate or combined treatment. GOLGA3 decreases after sirolimus treatment. AKR1C3 increases after combined treatment. These results are consistent with the mass spectrometry findings.

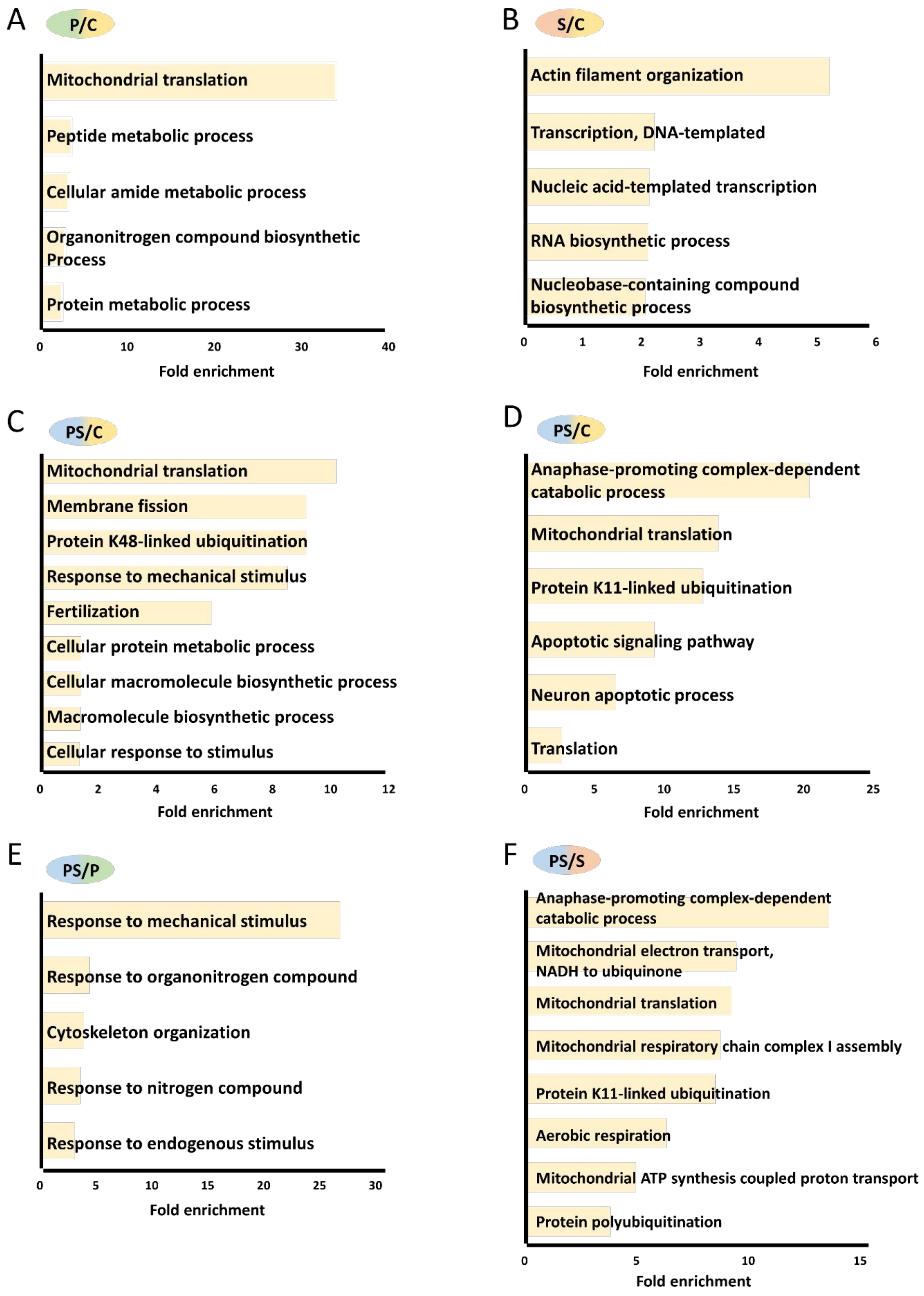

Fig. S4. DAVID analysis reveals the distinct impact of pyrvinium pamoate, sirolimus, and their combined treatment on the SK-N-DZ cell line. (A-F) Key terms identified through

DAVID analysis illustrate the differences in the effects of monotreatment (P/C and S/C) and combined therapy compared to control group (PS/C). Additionally, the influence of combined therapy compared to individual treatments (PS/P or PS/S) are also illustrated.

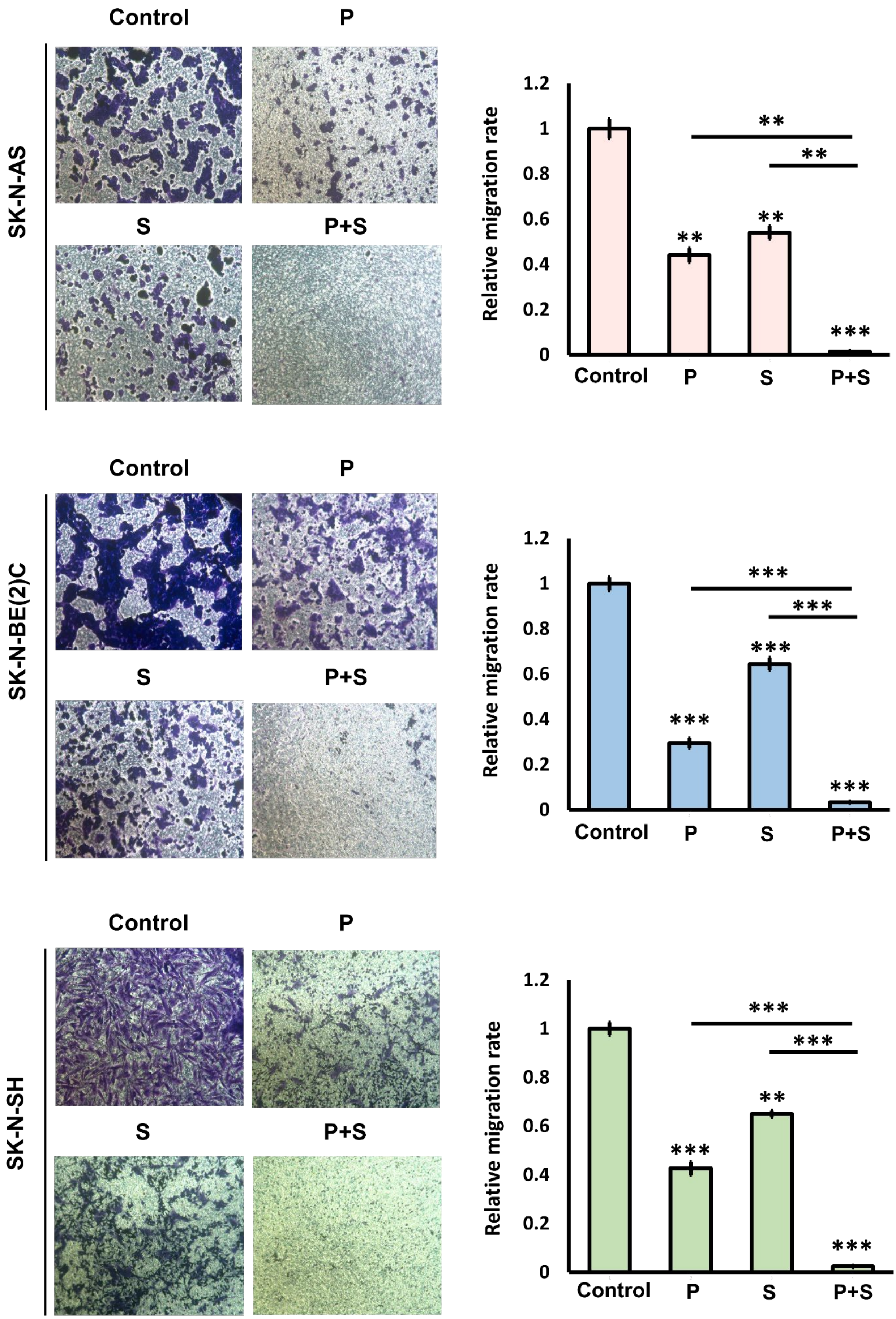
SK-N-AS
Control
P
S
P+S
Relative migration rate
1.2
1
0.8
0.6
0.4
0.2
0
**
**
**
**
***
SK-N-BE(2)C
Control
P
S
P+S
Relative migration rate
***
***
***
***
***
SK-N-SH
Control
P
S
P+S
Relative migration rate
***
***
**
***
***

Fig. S5. Combination treatment inhibits cell migration in SK-N-AS, SK-N-BE(2)C, and SK-N-SH neuroblastoma cell lines. Neuroblastoma cells were seeded into the upper chambers of Transwell inserts with 8 μm pore-size polycarbonate membranes. After 12 hours of incubation, cells that migrated to the lower side of the membrane toward medium with 10% FBS were fixed with methanol and stained with 0.5% crystal violet. For quantification, five random fields per insert were imaged under a light microscope. The stained areas (purple) indicating migrated cells were quantified using ImageJ software, and migration was expressed as a percentage relative to the control. All experiments were performed with triplicate with three biological and three technical replicates. Quantitative data are shown as bar graphs (mean ± SD).

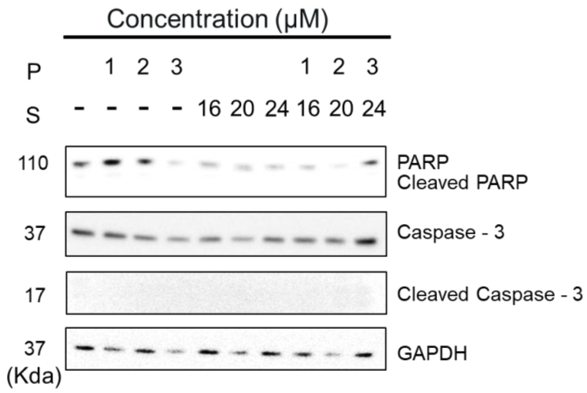

Fig. S6. Expression levels of apoptosis-related proteins in each treatment in SK-N-DZ cells.

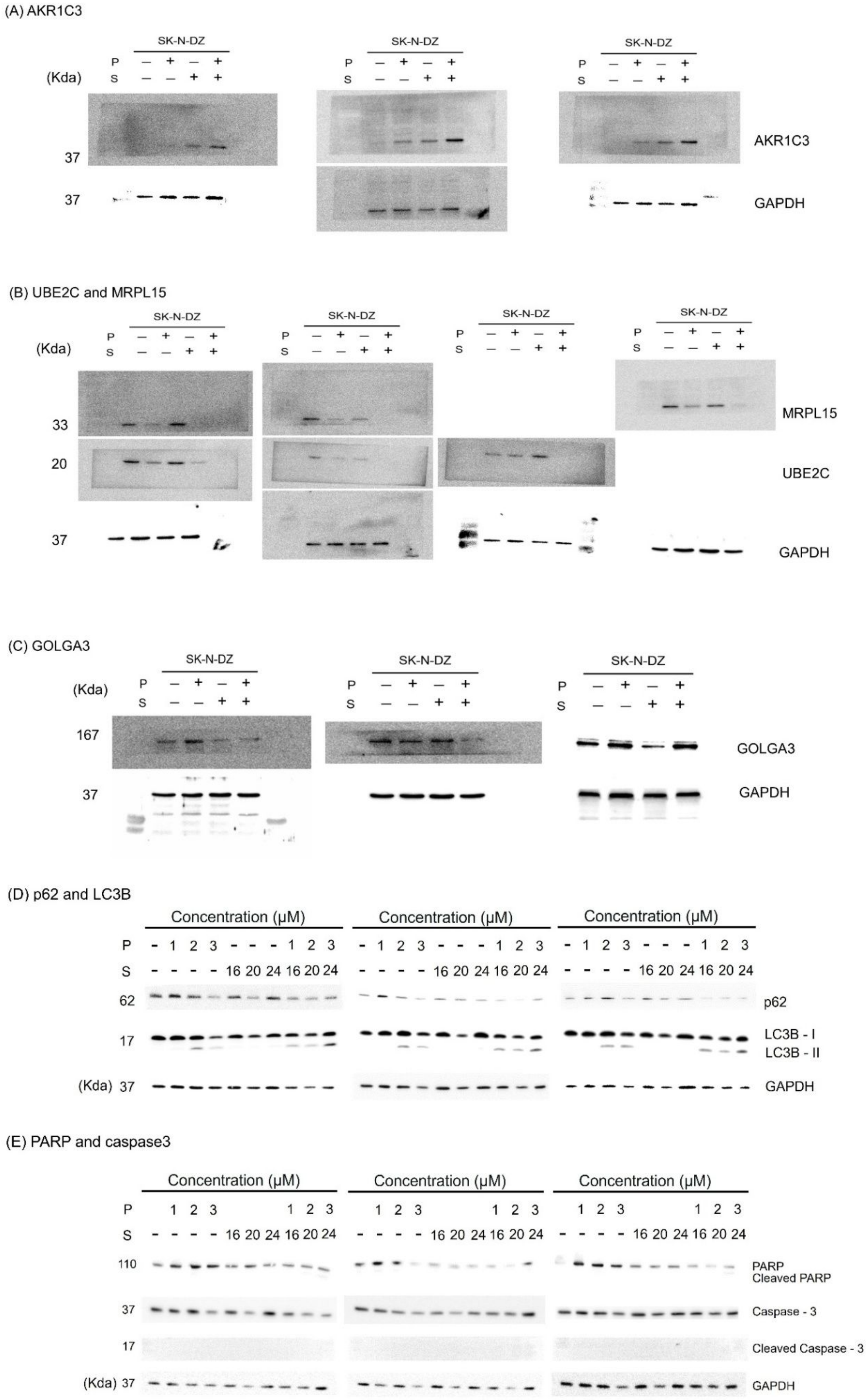

Fig. S7. Western blot original images.
